# Transcriptional induction of the heat shock protein B8 mediates the clearance of misfolded proteins responsible for motor neuron diseases

**DOI:** 10.1038/srep22827

**Published:** 2016-03-10

**Authors:** Valeria Crippa, Vito G. D’Agostino, Riccardo Cristofani, Paola Rusmini, Maria E. Cicardi, Elio Messi, Rosa Loffredo, Michael Pancher, Margherita Piccolella, Mariarita Galbiati, Marco Meroni, Cristina Cereda, Serena Carra, Alessandro Provenzani, Angelo Poletti

**Affiliations:** 1 Laboratory of Experimental Neurobiology, C. Mondino National Neurological Institute, Pavia, Italy; 2 Centre for Integrative Biology (CIBIO), Università degli Studi di Trento, Trento, Italy; 3 Dipartimento di Scienze Farmacologiche e Biomolecolari (DiSFeB), Centro di Eccellenza sulle Malattie Neurodegenerative, Università degli Studi di Milano, Milano, Italy; 4 Dipartimento di Scienze Biomediche, Metaboliche e Neuroscienze, Universita’ di Modena e Reggio Emilia, Modena, Italy; 5 Centro InterUniversitario sulle Malattie Neurodegenerative, Università degli Studi di Firenze, Genova, Roma Tor Vergata and Milano, Italy

## Abstract

Neurodegenerative diseases (NDs) are often associated with the presence of misfolded protein inclusions. The chaperone HSPB8 is upregulated in mice, the human brain and muscle structures affected during NDs progression. HSPB8 exerts a potent pro-degradative activity on several misfolded proteins responsible for familial NDs forms. Here, we demonstrated that HSPB8 also counteracts accumulation of aberrantly localized misfolded forms of TDP-43 and its 25 KDa fragment involved in most sporadic cases of Amyotrophic Lateral Sclerosis (sALS) and of Fronto Lateral Temporal Dementia (FLTD). HSPB8 acts with BAG3 and the HSP70/HSC70-CHIP complex enhancing the autophagic removal of misfolded proteins. We performed a high-through put screening (HTS) to find small molecules capable of inducing HSPB8 in neurons for therapeutic purposes. We identified two compounds, colchicine and doxorubicin, that robustly up-regulated HSPB8 expression. Both colchicine and doxorubicin increased the expression of the master regulator of autophagy TFEB, the autophagy linker p62/SQSTM1 and the autophagosome component LC3. In line, both drugs counteracted the accumulation of TDP-43 and TDP-25 misfolded species responsible for motoneuronal death in sALS. Thus, analogs of colchicine and doxorubicin able to induce HSPB8 and with better safety and tolerability may result beneficial in NDs models.

Different human diseases have been associated with an insufficient or an excessive intracellular response to proteotoxic stress. Proteins that lose the capability to acquire and/or to maintain the proper folding, after genetic mutations or damages in their structures (e.g.: specific amino acids modifications due to free radical exposure, etc.), become aberrantly folded or misfolded and can trigger a proteotoxic stress^1^,^2^. Misfolded proteins may oligomerize into aggregates that accumulate in cells. Misfolded proteins are potentially toxic by affecting several fundamental intracellular functions, either/both in their monomeric or/and in their aggregated forms, particularly in post-mitotic cells like neurons^3^,^4^. For these reasons, misfolded proteins are physiologically cleared from the cells by the protein quality control (PQC) system. The PQC system is composed of two main arms: the molecular chaperone, mainly represented by the heat shock proteins (HSPs)^5^,^6^,^7^ and the degradative pathways, including the proteasome, the autophagic response and the unfolded protein response (UPR)^8^,^9^. A specific degradative pathway for a given misfolded protein is selected by defined class of chaperones, with the assistance of co-chaperones^5^. These may determine whether a misfolded protein has to be refolded or degraded. We found that a specific chaperone, the small heat shock protein B8 (HSPB8), recognizes and promotes the removal of the misfolded proteins associated with motoneuron diseases (MNDs) (i.e. mutant SOD1 and a TDP-43 fragment in Amyotrophic Lateral Sclerosis (ALS); androgen receptor with an expanded polyglutamine tract (ARpolyQ) in Spinal and Bulbar Muscular Atrophy (SBMA)) promoting their autophagic removal from motoneurons^10^,^11^,^12^,^13^,^14^. HSPB8 acts in conjunction with the co-chaperone BAG3 (by complexing with a 2:1 ratio)^15^,^16^,^17^, a protein capable to interact with HSP70 bound to the ubiquitinating enzyme CHIP. After substrate ubiquitination, the autophagy receptor p62/SQSTM1 recognizes the cargo and mediates its insertion into the autophagosomes^13^,^14^,^18^. Our data showed that HSPB8 is a limiting factor in this process, since its overexpression enhances while its silencing reduces the intracellular clearance of all MNDs-associated misfolded proteins tested so far^9^,^12^,^13^,^19^. It must be noted that HSPB8 is highly expressed in the anterior horn lumbar spinal cord motoneurons that survive at end stage of disease in transgenic (tg) ALS-SOD1 mice, where it possibly acts as a protective agent against mutant SOD1 neurotoxicity^13^,^14^. Similar data have been reported in autopsy specimens of spinal cord of ALS patients^20^. Notably, in mouse spinal cord, the HSPB8 expression levels decline with age^13^, indicating that motoneurons may become more vulnerable to misfolded protein toxicity during aging, as a result of low levels of this pro-autophagic protein.

HSPB8 is robustly induced also in muscles of both tg ALS-SOD1 mice^1^,^19^,^21^ and knock-in (KI) AR113Q mice (KI SBMA mice, a model of SBMA)^9^,^22^. Muscle tissues have been found to be the target of misfolded protein toxicity both in ALS and SBMA^23^,^24^,^25^,^26^,^27^,^28^,^29^,^30^,^31^,^32^,^33^,^34^,^35^,^36^,^37^,^38^. In ALS and SBMA mice models, HSPB8 induction in muscle is accompanied by the induction of several other genes involved in the autophagic clearance of misfolded proteins, like p62/SQSTM1, LC3, BAG3 and BAG1^1^,^21^,^22^. HSPB8 also participates in the clearance of other misfolded proteins responsible for neurodegenerative diseases (NDs), such as two polyglutamine-containing proteins: mutant huntingtin responsible for Huntington Disease (HD) and ataxin-3 responsible for Spinal Cerebellar Ataxia 3 (SCA-3)^10^, as well as of beta-amyloid involved in Alzheimer’s disease (AD)^39^ and of alpha-synuclein involved in Parkinson’s disease (PD)^40^,^41^. In addition, HSPB8 has been found mutated in two NDs, Charcot-Marie-Tooth type 2 L disease and hereditary distal motor neuropathy type II (dHMNII)^42^,^43^. HSPB8 mutants lack their chaperone activity and impair lysosomal delivery of autophagosomes causing autophagic flux blockage^41^,^44^.

Collectively, these data prove that HSPB8 has a crucial activity for motor neuron (and possibly for muscle) function and viability and exerts a protective role against misfolded protein accumulation.

Based on these observations, in this study, we set up a high throughput screening (HTS) to identify commercially available FDA-approved small molecules and selected natural compounds capable to enhance HSPB8 expression in neuronal cells. The system takes advantage of the promoter region of the human *HSPB8* gene fused to firefly luciferase. We found several compounds that activates the HSPB8 promoter and two of these, colchicine and doxorubicin, were able to induce HSPB8 mRNA (and protein) expression. Interestingly, colchicine and doxorubicin also upregulated other proteins involved in the autophagic clearance of misfolded proteins such as the master regulator Transcription Factor EB (TFEB). The same two compounds were also able to greatly reduce the accumulation of misfolded species of TDP-43 and its 25 KDa fragment, whose accumulation is involved in sporadic forms of ALS (sALS).

## Results

### HSPB8 counteracts TDP-43 and TDP-25 misfolded species accumulation

**Figure 1:**
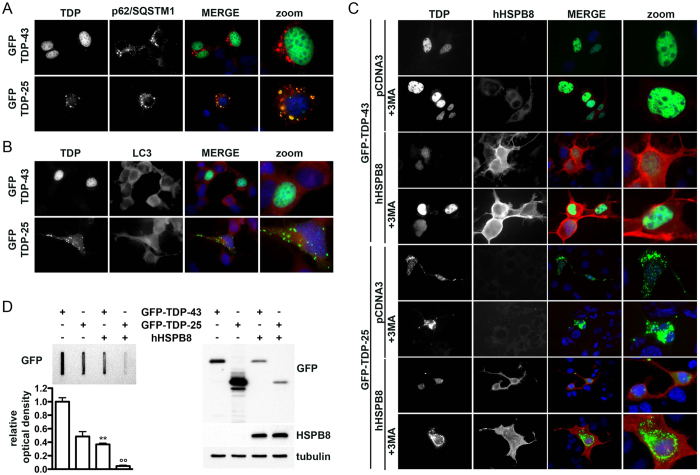
HSPB8 enhances the clearance of TDP-43 and TDP-25 (**A,B**) Immunofluorescence microscopy analysis (63x magnification and 2.5x zoom) of mouse motoneuronal NSC34 cells transiently overexpressing GFP-TDP-43 or GFP-TDP-25 plasmids. Cells were stained for the endogenous autophagic markers p62/SQSTM1 (**A**) and LC3 (**B**). Nuclei were stained with DAPI. (**C**) Immunofluorescence analysis of NSC34 cells transiently overexpressing GFP-TDP-43 or GFP-TDP-25 and pCDNA3 or hHSPB8. Where indicated, cells were treated with 10 mM 3-methyladenine (3-MA) for the last 48 hrs prior to fixation. DAPI: nuclei staining. RED: human transfected HSPB8. (**D**) Representative Filter Retardation Assay (FRA) and FRA quantification (left insets), and Western Blot (WB; right insets) of the PBS extracts of NSC34 cells transiently overexpressing the GFP-TDP-43 or GFP-TDP-25 and pCDNA3 or hHSPB8 plasmids. The histogram represents densitometric analysis of three independent replicates for each sample (**p < 0.01 vs GFP-TDP-43; °°p < 0.01 vs GFP-TDP-25).

The pro-degradative effects of HSPB8 have been proved to be efficient on different elongated polyQ containing proteins^9^,^10^,^45^ and on mutant proteins causing familial ALS forms (mutant G93A-SOD1 and a N-terminal fragment of TDP-43)^13^,^14^. We thus wanted to verify whether the protective activities of HSPB8 is not restricted to inherited NDs, but can also be extended to sporadic forms at least in the case of MNDs in which misfolded protein toxicity has been clearly implicated. To this purpose, we evaluated whether HSPB8 enhances the clearance of TDP-43 and its C-terminal fragment of 25 KDa (TDP-25), which is highly aggregation-prone due to the presence of a prion-like domain^46^. In fact, TDP-25 acts as seeding protein that sequesters into cytoplasmic insoluble aggregates endogenous wildtype TDP-43^47^, thereby affecting its subcellular localization and function and causing toxicity in ALS cell models. The TDP-25 fragment (TDP-25, residues 184–414) is aberrantly generated by caspase cleavage of TDP-43, possibly under stress condition^46^. The cleavage removes one of the two RNA Recognition Motifs (RRM) of TDP-43 and the Nuclear Localization Sequence (NLS), retaining the Nuclear Exportation Sequence (NES) and thus allowing TDP-25 mislocalization to the cytoplasm, a process that has been proposed to be part of the disease mechanism in sALS^46^,^47^. TDP-25 is mainly degraded via autophagy, while the TDP-43 is mainly cleared by the proteasome and chaperone-mediated autophagy (CMA) (even if also macroautophagy could be involved). Thus, we initially analysed the differential effect of HSPB8 on the two TDP-43 protein variants. As shown in Fig. 1A, the overexpression of the two proteins correlated with the formation of p62 bodies in immortalized motoneuronal cells, a clear index of autophagic flux blockage. In the case of TDP-43 the vast majority of the protein was found in the nuclei, while p62 bodies were localized in the cytoplasm. Therefore their appearance is an index of an overall induction of proteotoxic stress to the cells. In the case of TDP-25, the p62 bodies were adjacent to the TDP aggregates, but they did not physically sequester p62/SQSTM1. In both cases LC3, appeared with a diffuse distribution with some puncta indicative of the presence of autophagosomes (Fig. 1B). Thus, as expected, the autophagic clearance of the TDP-25 was impaired, allowing its accumulation into intracellular inclusions. The overexpression of HSPB8 promoted the clearance of the TDP-43 protein variants (Fig. 1C). In fact, the chaperone reduced the concentration of TDP-43 in the nucleus and its overexpression resulted in a robust reduction of intracellular TDP-25 aggregates from the cytoplasm. The HSPB8 prodegradative activity was abolished by the autophagy inhibitor, 3-MA (Fig. 1C). The data obtained in immunofluorescence microscopy, were also confirmed in filter retardation assay (FRA) and in Western Blot (WB) analysis (Fig. 1D), in which we showed that HSPB8 overexpression robustly reduced the overall accumulation of both TDP-43 and TDP-25 insoluble species retained in cellulose acetate membrane (FRA and FRA quantification, Fig. 1D, left insets) and TDP-43 and TDP-25 soluble species detected in WB (WB, Fig. 1D, right insets).

### Development of the cellular system for the HTS of HSPB8 inducers

**Figure 2:**
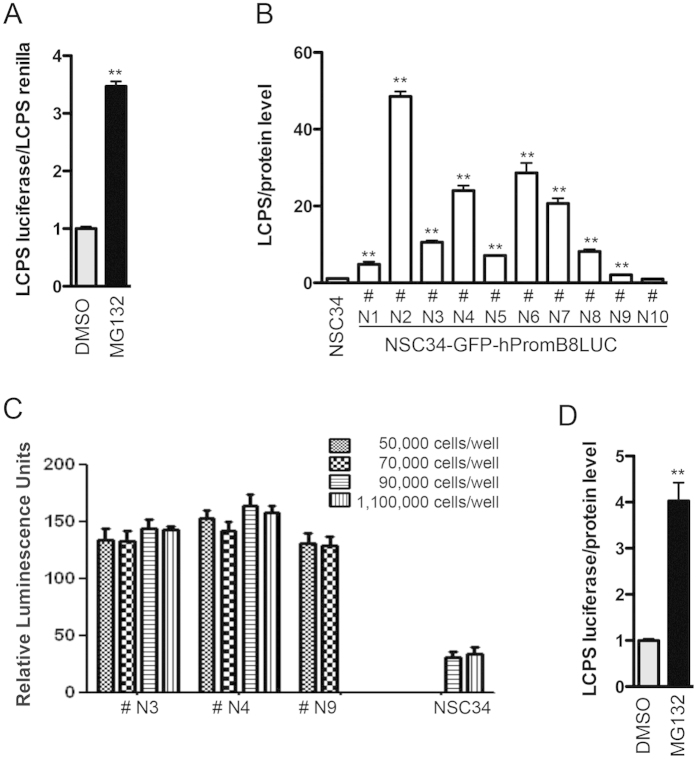
Development of the cellular system for the screening of the HSPB8 promoter modulators (**A**) Validation of the GFP-hPromB8LUC plasmid. NSC34 cells were transiently transfected with GFP-hPromB8LUC and pRL-TK plasmids. 24 hrs after transfection, cells were treated with DMSO or 10 μM MG132 for the last 16 hrs prior to luminescence analysis. The histogram represents the Luminescence Counts Per Second (LCPS) of the inducible firefly luciferase normalized by the LCPS of renilla luciferase of six independent replicates (**p < 0.01 vs DMSO). (**B**) Clones selection of NSC34-GFP-hPromB8LUC (#Ns) stably transfected cells. Different #Ns clones were analysed for their luminescence levels, 48 hrs after plating. NSC34 untransfected cells were used as negative control. The histogram represents the firefly luciferase LCPS mean normalized by the total protein level mean of six independent replicates for each clone (**p < 0.01 vs NSC34 untransfected cells). (**C**) Optimization of cell number and signal to background ratio for HTS assay. Raw data of relative luminescence units detected by NSC34 clones after 24 hrs seeding at the indicated cell number. SD refers to two independent experiments. (**D**) Validation of the #N4 selected clone. NSC34-GFP-hPromB8LUC #N4 cells were analysed for their ability to respond to MG132 treatment. 24 hrs after plating, cells were treated with DMSO or 10 μM MG132 for the last 16 hrs prior to luminescence analysis. The histogram represents the firefly inducible luciferase LCPS mean normalized by the total protein level mean of six independent replicates (**p < 0.01 vs DMSO).

According to these and reported data^10^,^11^,^12^,^13^, we set up an assay that could reveal the up-regulation of HSPB8 mRNA expression in neuronal cells, and that was amenable to high-throughput screening (HTS). The aim was to identify an inducer of HSPB8 expression after screening a library of small molecules. To this purpose, we amplified the human genomic DNA region upstream of the transcription initiation site of the human HSPB8 gene. This region was cloned into a luciferase-based expression vector to drive luciferase expression under the regulation of the exogenous HSPB8 promoter. The vector also contained a cDNA sequence coding for the GFP under the control of the CMV promoter to facilitate the screening of positive stably transfected clones. Initially, in transiently transfected immortalized motoneuronal (NSC34) cells, we tested whether the human HSPB8 promoter was activated by proteasome inhibition, which we already proved to activate transcription of HSPB8 promoter and to induce HSPB8 expression in different models^11^,^12^,^13^. The treatment with MG132, a potent inhibitor of the chymotryptic activity of the proteasome^48^,^49^, significantly induced the transcriptional activity of the human HSPB8 promoter in NSC34 cells transiently transfected cells (Fig. 2A; +MG132 mean = 3.47 ± 0.24). We next transfected NSC34 cells and selected stably transfected clones (#Ns NSC34-GFP-hPromB8LUC cells) both for their resistance to Geneticin (G418) and for the GFP fluorescence. Positive clones have been characterized by the expression level of luciferase under basal condition (Fig. 2B). Clones #N3, #N4 and #N9, characterized by low to medium levels of luciferase expression were selected to obtain a larger signal to background window in the case of an increased expression of the luciferase protein during the screening process. These clones were further screened during HTS assay optimization to determine best cell density and signal to background ratio of luciferase activity. Although with a marginal improvement, the best condition of low background luciferase signal was obtained using #N4 clone cells at a density of 9^*^10^4^ cells/well (Fig. 2C).

The quantification of the luciferase activity for the #N4 clone in basal condition or after treatment with MG132 confirmed that the human HSPB8 promoter integrated into the genome of NSC34-GFP-hPromB8LUC cells was still able to respond to proteasome inhibition (Fig. 2D).

### Hits identification and validation

**Figure 3:**
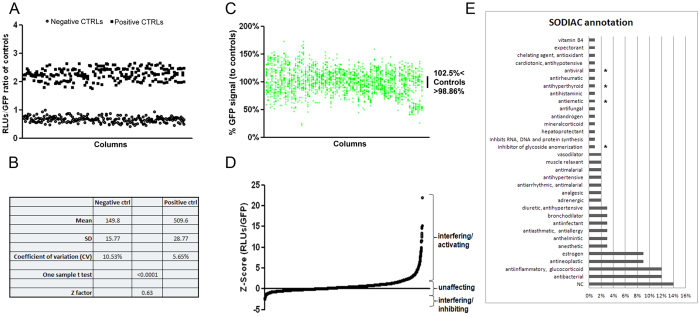
Primary screening parameters and ranking of the 2,000 library compounds. (**A**) Distribution of positive (MG132) and negative (DMSO) controls according to firefly units normalized to GFP signal. #N4 cells were treated with 5 μM of MG132 or DMSO for 24 hrs in two columns (1–2 and 23-24, respectively) of each 384-well plate ran (n = 25). (**B**) Parameters calculated from positive and negative controls validating robustness of the screening. (**C**) Distribution of GFP signals relative to each compound tested; values near to 100% indicate un-perturbing activity of compound on cellular or GFP-associated metabolism. (**D**) Plot of compounds ranked according to progressive Z score values (compounds are listed in Supplementary Table 2). (**E**) Chemical ontology (SODIAC) annotation according to the recognized bioactivity of HSPB8 activating compounds. *indicates less than 1% over-representation; NC = non classified.

We set up the HTS using MG132 as positive control with respect to untreated cells, used as negative control (Fig. 3A). In this workflow, the firefly luciferase signal was normalized with relative GFP fluorescence. The 5 μM MG132 treatment condition allowed to obtain a signal to background window of 3.4, a mean CV value among controls of 8.09% and a Z-factor of 0.63, indicating good parameters and reliability of the HTS assay (Fig. 3B). In Fig. 3C the distribution of green signal intensity is shown according to treatment of NSC34-GFP-hPromB8LUC [#N4] cells with the 2,000 compounds screened (list of compounds, Supplementary Table 1); the standard deviation of controls was as low as 1.82% (range from 102.5% to 98.86%). The plot of compounds (Fig. 3C), indicates that almost 56% of all molecules tested influenced GFP expression after 24 hrs treatment in these cells.

Therefore, to avoid nonspecific effect on luciferase expression, we normalized luminescence units with GFP fluorescence levels, and the results of primary screening were plotted according to progressive Z-score values (Fig. 3D). We classified compounds as activating (554, 27.7%), unaffecting, and interfering/inhibiting (48, 2.4%) (Fig. 3D and Supplementary Table 1). Within the activating compound’s set and among the first 246 (12.3%) with a positive effect on luciferase expression, first 18 were confirmed in duplicate as top-ranked compounds (Supplementary Table 2). By using the SODIAC chemistry ontology (http://ontochem.de/it-solutions/what-we-offer/sodiac.html), we classified the top scored compound according to their recognized/noted bioactivity and found categories prominently represented (in order of significance) by estrogen (9%), antineoplastic (9%), anti-inflammatory (12%), and antibacterial (12%) compounds (Fig. 3E).

### Dose-response analysis of the 18 selected hits

**Figure 4:**
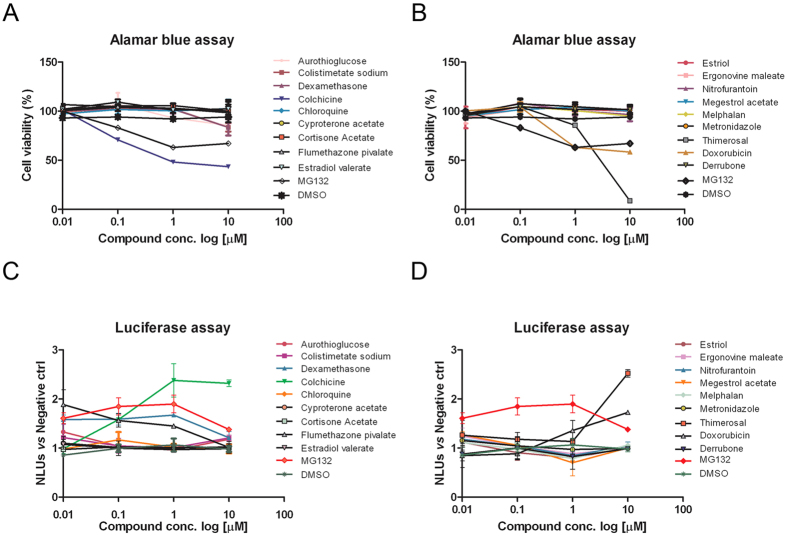
Counterscreening of 18 hits by dose-response effects. (**A,B**) NSC34 #N4 cells were treated for 24 hrs with four doses of indicated compounds. Relative cytotoxic activity was evaluated by Alamar blue assay following manufacture’s instruction. (**C,D**) Relative luminescence units detected in cells treated as in (**A,B**), normalized to cell viability (n = 2).

**Figure 5:**
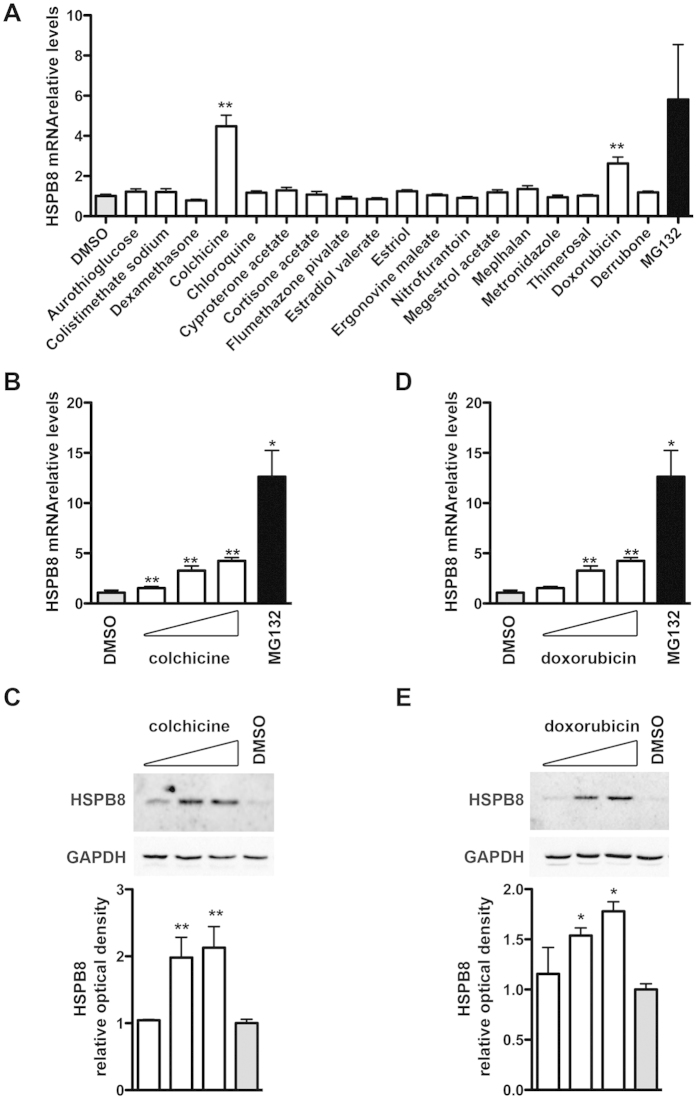
Hits validation on endogenous HSPB8 expression in human neuronal cells (**A**) Q-PCR analysis of HSPB8 mRNA levels performed on SH-SY5Y cells. 24 hrs after plating, cells were treated for the last 24 hrs prior to extraction with 1 μM of different compounds or with DMSO (negative control) or 10 μM MG132 (positive control). HSPB8 mRNA levels were normalized with RplP0 mRNA levels (relative levels); histogram represents the analysis of 4 independent replicates (**p < 0.01 vs DMSO). (**B–E**) Dose-dependent response analysis of colchicine (**B,C**) and doxorubicin (**D,E**) treatments performed on SH-SY5Y cells treated with 100 nM, 500 nM or 1 μM of colchicine or doxorubicin, and with DMSO or 10 μM MG132 for 24 hrs. (**B,D**) Q-PCR analyses of HSPB8 mRNA levels. The histograms represent the HSPB8 mRNA relative levels mean of 4 independent replicates (*p < 0.05 vs DMSO; **p < 0.01 vs DMSO). (**C,E**) WB and WB quantification of HSPB8 protein levels. The histograms represent densitometric analysis of three independent replicates for each sample (*p < 0.05 vs DMSO; **p < 0.01 vs DMSO).

The secondary screening has been performed as dose-response curve at 24 hrs in #N4 cells and by normalizing relative luminescence signals on absorbance data of the Alamar blue assay (Fig. 4A,B), to correct the cytotoxicity exerted by different compounds. MG132 was used as positive control. The cell viability resulted slightly affected by the majority of agents tested in the range of doses used, although cytotoxic effects of some compounds were prevalent at 10 μM, i.e. of thimerosal, doxorubicin, and colchicine. As shown in Figs 4C,D and 5 out of 18 hits, represented by dexamethasone, flumethazone, colchicine, thimerosal, and doxorubicin displayed highly significant effect (P < 0.001) at 1 μM; 4 out of them, i.e. colistimetate, megestrol, melphalan, and metronidazole showed a significant effect (P < 0.05) at 1 μM; while cyproterone and nitrofurantoin compounds displayed a slight dose-response trend at the doses used, accounting for a total of 11 compounds (61%) globally validating approximately 0.5% of hits from the primary screening.

### Validation of the hits for their ability to induce endogenous HSPB8 expression

We next determined whether the active compounds selected from our primary screening were also capable to induce the expression of the endogenous HSPB8 gene. In order to preserve the same promoter sequence adopted in the HTS assay and since NSC34 cells are of mouse origin, we used human derived neuroblastoma cells (SH-SY5Y), which are characterized by a neuronal-like phenotype and can be differentiated to neuronal cells with retinoic acid^50^. Cells were treated with the selected compounds for 24 hrs to test their effect on the total amount of HSPB8 mRNA. The results have shown that only colchicine and doxorubicin were able to robustly upregulate the expression of the endogenous human HSPB8 in neuroblastoma cells (Fig. 5A). Induction was approximately of 3–5 folds over control untreated cells. SH-SY5Y cells lack the estrogen receptors; in line, the estrogenic compounds identified in our primary screening were unable to induce HSPB8 in these cells.

Therefore, we focused on colchicine and doxorubicin as the best candidates to induce HSPB8 expression and protein production in neuronal cells. We initially performed a dose-response analysis to evaluate the optimal concentration required for colchicine and doxorubicin treatments to significantly induce HSPB8 expression. The results showed that colchicine was active at 100 nM (Fig. 5B,C), while doxorubicin was active at doses slightly higher of 500 nM (Fig. 5D,E). In both cases, a clear dose-response effect was observed both by measuring HSPB8 mRNA level by Q-PCR (Fig. 5B,D) or the protein levels using a specific anti-human HSPB8 antibody by western blot (Fig. 5C,E).

### Colchicine and doxorubicin effects on the heat shock response

**Figure 6:**
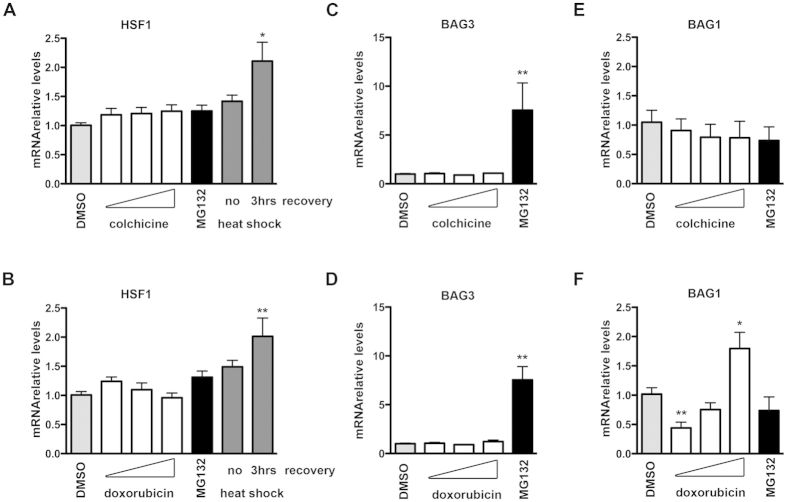
Analysis of the heat shock response after colchicine and doxorubicin treatments (A–F) Q-PCR analyses of HSF1 (A,B), BAG3 (C,D) and BAG1 (E,F) mRNA levels performed on SH-SY5Y cells treated for the last 24 hrs prior to extraction with 100 nM, 500 nM or 1 μM of colchicine (A,C,E) or doxorubicin (B,D,F), and with DMSO or 10 μM MG132. Where indicated, cells where heat shocked for 1hr at 42 °C, and collected after the shock (no recovery) or after a recovery period of 3hrs at 37 °C. Analysed mRNA levels were normalized with RplP0 mRNA levels and expressed as relative levels mean of 4 independent replicates (*p < 0.05 vs DMSO; **p < 0.01 vs DMSO).

We next evaluated whether the two compounds modulated the heat shock response in neuronal SH-SY5Y cells by measuring the expression of the Heat Shock Factor 1 (HSF1) and of two classical nucleotide exchange factors (NEFs) assisting the ATP-dependent activities of HSP70 (and CHIP)^9^,^13^,^15^,^21^,^51^,^52^,^53^, BAG1 and BAG3. These NEFs control the alternative routing of HSP70/CHIP associated misfolded proteins to proteasome (BAG1) or autophagy (BAG3 in association with HSPB8); in particular, BAG3, which forms a stoichiometric complex with HSPB8, regulates the prodegradative action of HSPB8^13^,^15^,^21^. While our cells positively responded to control stimuli by increasing two-fold HSF1 expression during recovering time after heat shock (Fig. 6A,B), colchicine and doxorubicin were not able to change the basal expression level of HSF1 (Fig. 6A). Colchicine also did not modify BAG3 (Fig. 6C) or BAG1 (Fig. 6E). Doxorubicin behaved similarly (Fig. 6D,F), but having a bi-phasic effect on BAG1. Taken together these data show that these drugs do not stimulate the HSF1-dependent stress response.

### Colchicine and doxorubicin effects on the autophagic pathway

**Figure 7:**
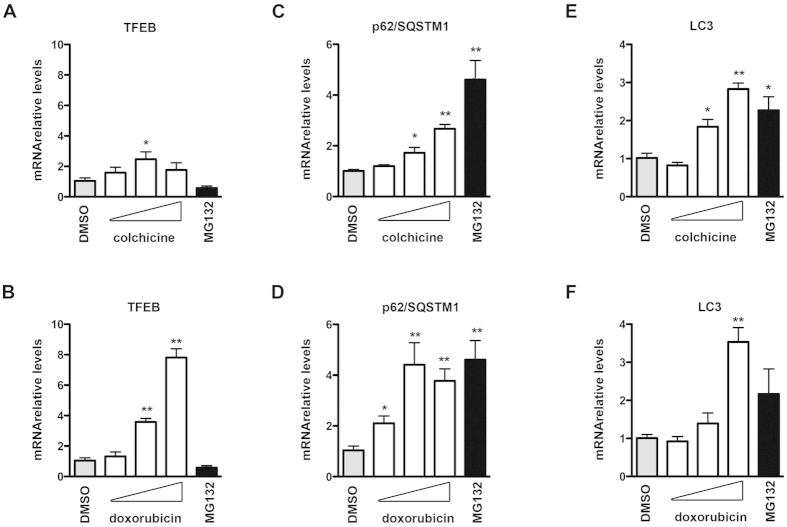
Colchicine and doxorubicin effects on the autophagic pathway (A–F) Q-PCR analyses of TFEB (A,B), p62/SQSTM1 (C,D) and LC3 (E,F) mRNA levels performed on SH-SY5Y cells treated as described in Fig. 6. The histograms represent analysed mRNA relative levels mean of 4 independent replicates (colchicine treatment, **A**, **C** and **E**; doxorubicin treatment, **B, D** and **F**) (*p < 0.05 vs DMSO; **p < 0.01 vs DMSO).

**Figure 8:**
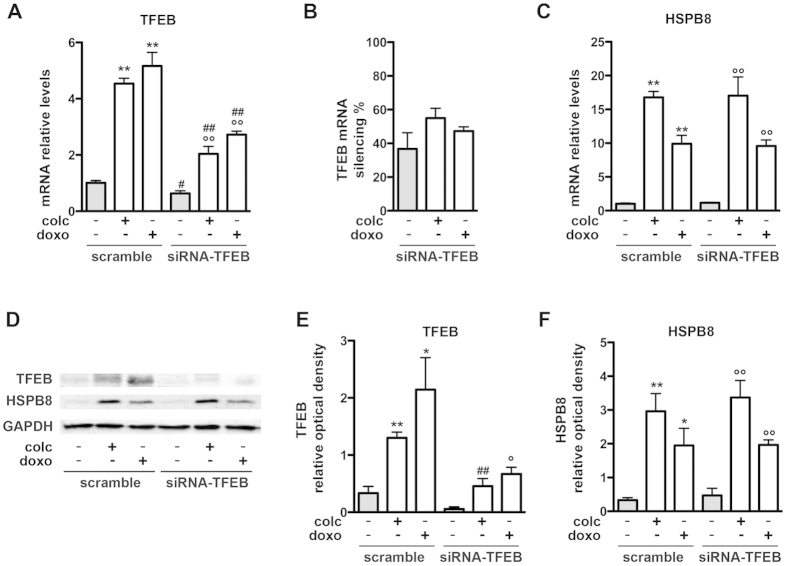
Colchicine and doxorubicin effects on HSPB8 expression are TFEB-independent (A–F) Analysis of the TFEB silencing on HSPB8 expression performed on SH-SY5Y cells transfected with an ON-TARGETplus TFEB siRNA or scramble siRNA, and treated with 500 nM of colchicine or doxorubicin, and with DMSO for 24 hrs. (A) Q-PCR analyses of TFEB mRNA levels. TFEB mRNA relative levels were normalized on the levels of RplP0 and expressed as mean of 4 independent replicates (**p < 0.01 vs scramble/DMSO; °°p < 0.01 vs siRNA-TFEB/DMSO, ^#^p < 0.05 and ^##^p < 0.01 vs treatment-corresponding scramble samples). (**B**) The histogram represents the quantification of the TFEB silencing (expressed as percentage). (**C**) Q-PCR analyses of HSPB8 mRNA levels (**p < 0.01 vs scramble/DMSO; °°p < 0.01 vs siRNA-TFEB/DMSO). (**D**) WB and WB quantification of TFEB (**E**) and HSPB8 (**F**) protein levels. The histograms represent densitometric analysis of three independent replicates for each sample (*p < 0.05 and **p < 0.01 vs scramble/DMSO; °p < 0.05 and °°p < 0.01 vs siRNA-TFEB/DMSO, ^##^p < 0.01 vs treatment-corresponding scramble samples).

To better characterize the molecular mechanisms of colchicine and doxorubicin in SH-SY5Y cells, we also tested the possibility that the two compounds may induce the up-regulation of genes involved in the autophagic clearance of misfolded proteins. Very interestingly, we found that both colchicine and doxorubicin were able to induce the expression of the autophagy “master” gene regulator TFEB. The effect of colchicine was detectable only at 500 nM (Fig. 7A) while the induction of TFEB by doxorubicin was pronounced at the two higher doses utilized (Fig. 7B). Accordingly, the two compounds induced the expression of the autophagy markers p62/SQSTM1 and LC3 even if with different efficacies (Fig. 7C–F). To ascertain whether TFEB is the mediator of the induction of observed after colchicine and doxorubicin treatment, we evaluated the effects of the two compounds in cells in which TFEB was silenced with a specific siRNA. We obtained a 40% reduction of TFEB expression after siRNA-TFEB treatment; the latter was still efficient in reducing the upregulation of TFEB mRNA and protein upon exposure to colchicine or doxorubicin (Fig. 8A, compare scramble with siRNA-TFEB and 8B, silencing quantification). The induction of achieved with colchicine and doxorubicin was similar in control and TFEB-depleted cells (Fig. 8C), suggesting that TFEB is not the key factor responsible for the “de novo” synthesis of HSPB8 upon treatment with colchicine or doxorubicin. These data were also confirmed by WB analysis of TFEB and HSPB8 protein levels (Fig. 8D and quantification in Fig. 8E,F).

### Colchicine and doxorubicin effects on TDP-43 and TDP-25 misfolded species accumulation

**Figure 9:**
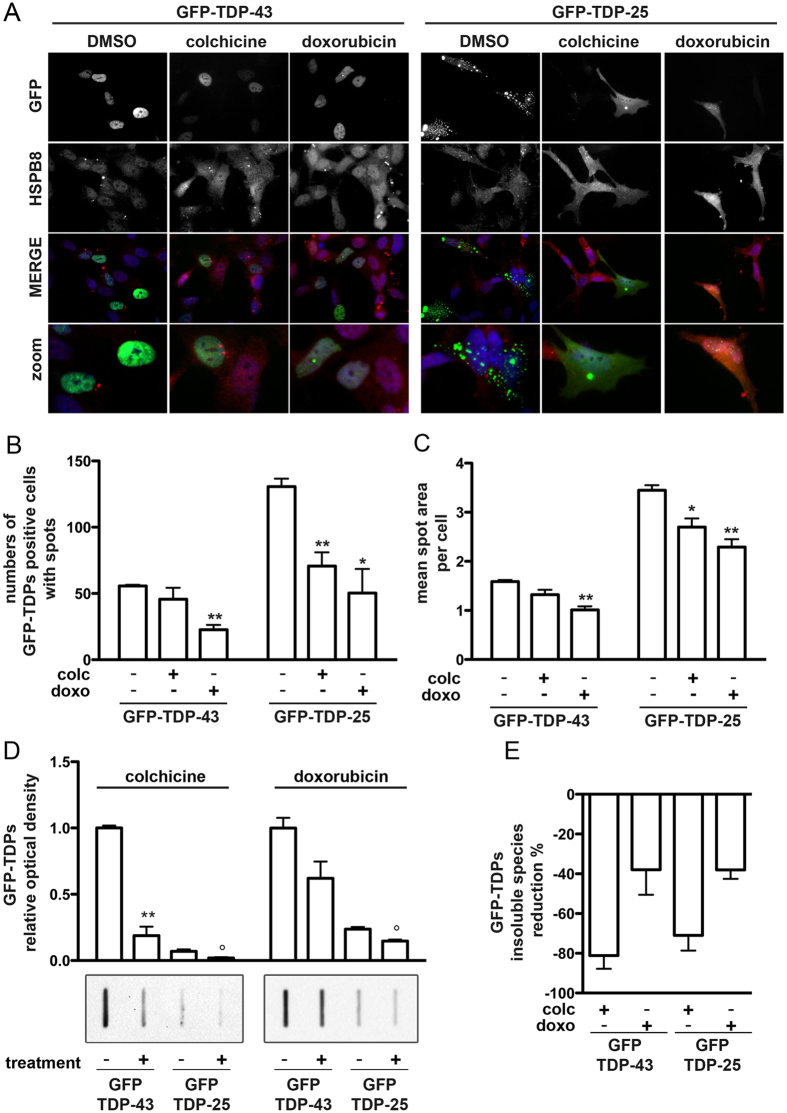
Colchicine and doxorubicin effects on TDP-43 and TDP-25 aggregation (A) Representative immunofluorescence microscopy analysis (63x magnification and 2.5x zoom) performed in SH-SY5Y cells transiently transfected for 24 hrs with GFP-TDP-43 or GFP-TDP-25 plasmids and then treated for 24 hrs with 1 μM colchicine, doxorubicin or DMSO. Left panels: GFP-TDP-43; right panels: GFP-TDP-25. DAPI: nuclei staining. RED: HSPB8. (**B**) Quantification of GFP-TDP-43 and GFP-TDP-25 positive cells with GFP-spots using Operetta Imaging System and Harmony software analysis (Perkin Elmer) on living SH-SY5Y cells transiently transfected and treated as described in A. Spots are defined with a range of size from 1 to 10 μm^2^ in SH-SY5Y treated as in A. SD refers to four independent replicates in HCS. (*p < 0.05 and **p < 0.01 vs untreated control). (**C**) Quantification of spots area in GFP-TDP-43 and GFP-TDP-25 positive SH-SY5Y cells after drugs treatment obtained by Harmony software analysis (Perkin Elmer).(n = 4). (*p < 0.05 and **p < 0.01 vs untreated control). (**D,E**) Representative FRA and FRA quantifications of the PBS extracts of SH-SY5Y cells transiently overexpressing the GFP-TDP-43 or GFP-TDP-25 plasmids treated or not with 500 nM of colchicine (left insets) or doxorubicin (right insets) the day after transfection, for the last 24 hrs prior to extraction. (**D**) The histograms represent densitometric analysis of three independent replicates for each sample (**p < 0.01 vs untreated GFP-TDP-43; °p < 0.05 vs untreated GFP-TDP-25). (**E**) The histogram represents the quantification of the GFP-TDPs insoluble species reduction percentage after treatment with colchicine or doxorubicin.

Finally, we evaluated whether colchicine and doxorubicin were able to reduce the accumulation of TDP-43 and its C-terminal fragment TDP-25 associated with sporadic forms of ALS. In SH-SY5Y transfected cells, fluorescence microscopy initially confirmed that both drugs increased the intracellular levels of endogenous HSPB8 (Fig. 9A). In addition, we found that very few GFP-TDP-43 expressing cells containing GFP-positive puncta were present (Fig. 9A, left panels). Conversely, in GFP-TDP-25 expressing cells we observed several cytoplasmic aggregates (Fig. 9A, right panels) that were almost completely removed by colchicine or doxorubicin treatments. Moreover, both colchicine and doxorubicin modified the overall levels of GFP-TDP-43 and of GFP-TDP-25 (Fig. 9A). In living SH-SY5Y cells, we carefully quantified the accumulation of GFP-TDP-43 and GFP-TDP-25 aggregates, here defined as “GFP-spots” (range of size from 1 to 10 μm^2^), using Operetta Imaging System, and Harmony software analysis (Fig. 9B,C). In GFP-TDP-43 transfected cells, only doxorubicin reduced the number of cells with “GFP-spots” (Fig. 9B); but, both drugs decreased the mean size of the spots (Fig. 9C). In cells expressing GFP-TDP-25, we found that both drugs were able to reduce the number of cells positive for “GFP-spots” (Fig. 8B) and to decrease the spots size (Fig. 9C). Next, we quantified the effects of the two compounds on the total amount of GFP-TDP-43 and GFP-TDP-25 insoluble species in FRA. Very interestingly, the treatment with colchicine resulted in a great reduction of insoluble species generated both by GFP-TDP-43 and by GFP-TDP-25 (Fig. 9D), while doxorubicin was particularly active on the GFP-TDP-25 fragment (Fig. 9D). We estimated the percentage of reduction in the accumulation of the two TDP-43 variants and found that the intensity of the effects exerted by the two compounds on the full length or on the fragment of TDP-43 were much more intense in the case of colchicine than in the case of doxorubicin (Fig. 9E).

## Discussion

In NDs characterized by the formation of inclusions containing misfolded proteins, the toxicity associated with these proteins can be counteracted by the activation of the PQC system. We previously characterized a specific member of the chaperone family, HSPB8, which is highly expressed in affected muscles of tg ALS mice and knock-in SBMA male mice models^9^,^19^,^22^. HSPB8 is also highly expressed in the spinal cord motor neurons of tg ALS mice that survive to mutant SOD1 toxicity at end stage of disease^13^,^14^ and in spinal cord of ALS patients^20^. HSPB8 has a potent pro-degradative activity for the elongated ARpolyQ involved in SBMA^11^,^12^,^17^,^45^, as well as for the mutant SOD1 and a C-terminal truncated fragment of TDP-43 responsible for different forms of fALS. The importance of HSPB8 is underlined also by its prodegradative activity on other misfolded proteins, such as htt in HD, ataxin-3 in SCA-3 and beta-amyloid in AD^10^,^39^.

Here, we demonstrated that HSPB8 also prevents the accumulation of misfolded species generated by TDP-43 and, especially, its 25 KDa fragment (TDP-25, involved in most sALS and FLTD forms). These species are known to exert their toxicity by mislocalizing TDP-43 from nucleus to cytoplasm^54^, where they aggregate entrapping rightly folded TDP-43, thereby reducing its nuclear functions^47^,^55^,^56^. The pro-degradative activity of HSPB8 has been associated with its capability to facilitate and enhance the autophagic removal of misfolded species by acting in conjunction with the HSP70/HSC70 co-chaperone BAG3 and CHIP^13^,^14^,^18^, as well as to counteract autophagic flux blockage induced by misfolded mutant proteins^9^,^12^. Therefore, with the aim to test the effects of HSPB8 induction for therapeutic purposes, in this study we set up a novel motoneuronal cell system to perform a HTS of small molecules to identify inducers of HSPB8 expression.

Our cell system is based on the stable expression of the luciferase cDNA driven by the human HSPB8 gene promoter in immortalized motor neurons^12^,^13^. We found several compounds that were able to activate the HSPB8 promoter as evaluated by the induction of luciferase activity in the transcriptional reporter assay. Combining their activities with their safety, we selected few candidates that were further tested for their power to enhance the expression of the endogenous HSPB8 in neuronal cells.

Some compounds able to induce HSPB8 transcription are estrogenic drugs^57^. However, neuronal SH-SY5Y cells do not express the two isoforms of estrogen receptor (ERalpha and ERbeta) and are thus unresponsive to these drugs. This represents an obvious limitation for the potential use of estrogenic compounds to enhance HSPB8 expression for therapeutic purposes, since their activity, in the specific neuronal population affected in ALS or FLTD, strictly depend on the brain distribution of the ERs, which may jeopardize the protective effects of the drugs. Additionally, we observed that 14% out of compounds has not obvious annotation of biological activity, as the case of derrubone (our first hit) of which some recent reports have highlighted a molecular chaperone activity^58^,^59^, further supporting the validity of our approach.

In the secondary screening on SH-SY5Y, we identified two compounds that robustly up-regulated, and in a dose-dependent manner, the expression of the human HSPB8: colchicine and doxorubicin. These compounds did not modify the expression of HSF1, a master regulator of the stress response, and a direct target of HSF1, BAG3^60^. Colchicine did not induce the expression of BAG1 while doxorubicin exerted a positive effect^61^, supporting the idea that doxorubicin may also activate a proteasomal response^51^. Conversely, both colchicine and doxorubicin positively regulated the expression of TFEB, the master regulator of the autophagic process, and of p62/SQSTM1 and LC3 that are specifically required to sustain the autophagic flux. Finally, both drugs counteracted the accumulation of both TDP-43 and TDP-25 misfolded species responsible for motoneuronal death in sALS.

It is interesting to note that both colchicine and doxorubicin are approved by FDA for the treatment of human diseases; but their safety and side effects are very different. Doxorubicin is a chemotherapic agent used in different cancer type (e.g. blood cancers, solid tumors and in sarcomas)^62^. The mechanism of action of doxorubicin is based on its property to intercalate and bind to DNA, inhibiting its replication by blocking the topoisomerase II and triggering apoptosis^63^,^64^. Additionally, it can activate an autophagic response that facilitates cell survival^65^. Doxorubicin needs to be administered intravenously and, unfortunately, when used chronically, is characterized by severe side effects, the most relevant being linked to heart damages, leading to cardiomyopathy^62^,^66^. Indeed the prolonged, doxorubicin-induced, autophagic and proteasomal response has been addressed as a co-cause of cardiomyocyte death^67^; however, increased acute autophagy with co-administration of rapamycin and doxorubicin rescued doxorubicin toxicity^68^.

Conversely, colchicine is a safer drug as compared to doxorubicin. Colchicine acts mainly by inhibiting microtubule polymerization during mitosis as a spindle poison^69^,^70^; with this activity colchicine blocks neutrophil motility and activity, and it is characterized by an anti-inflammatory activity. Because of that, colchicine is commonly used in gout treatment^71^,^72^, and less frequently, in pericarditis and recurrence of atrial fibrillation after cardiac tissue ablation^73^,^74^. Colchicine is administered orally, and it is usually well tolerated at low doses and shows limited adverse effects at high doses particularly on the gastrointestinal tract^73^. A potential limitation of colchicine has been observed in some patients with advanced renal failure exposed to long-term (prophylactic) oral colchicine treatment, in which a low fraction of colchicine is not metabolized and not removed by hemodialysis, causing cumulative toxicity that resulted in neuromyopathy^75^,^76^,^77^. Interestingly, high doses of colchicine combined with starvation enhance the autophagy flux in skeletal muscle of normal mice^78^. Surprisingly, the same approach, used to induce autophagy in tgALS mice, correlated with a reduced autophagic flux, possibly because of the presence of an excess of the misfolded SOD1 in skeletal muscle of these mice^78^. The study correlated the reduced autophagic flux with the fact that in skeletal muscle expressing mutant SOD1 there is an increased activity of caspase-3, which targets several key proteins involved in autophagy, such as Beclin-1, an important protein that bridges autophagy with apoptosis^78^. Thus, an altered autophagy activity can be at the basis of mutant SOD1 (and of other misfolded proteins) toxicity in this tissue^9^,^19^,^21^,^22^. Unfortunately, no treatments with colchicine alone were tested to clarify if this effect has to be ascribed to the effect of starvation or the combined factors. Obviously, as a proof of principle, the analysis of colchicine effect (and possibly doxorubicin) in animal models (both wt and tg models of NDs) will also confirm whether the drug is able to induce HSPB8 expression in skeletal muscle and in neurons (including motoneurons) affected by proteotoxicity of misfolded proteins. These studies will also clarify whether colchicine (or doxorubicin) ameliorates clinical signs of disease and whether it is capable to extend survival of affected mice. This will allow to design analogs of the two HSPB8 inducers characterized by better safety and tolerability profiles.

## Methods

### Chemicals

Dimethyl sulfoxide (DMSO), Colchicine, Doxorubicin and MG132 were obtained from Sigma-Aldrich (St. Louis, MO, USA), 3-Methyladenine (3-MA) was obtained from Selleckchem (Munich, Germany), Geneticin (G418) was from Euroclone (Pero, Milan, Italy). The commercial library of 2,000 compounds was from MicroSource (SPECTRUM Collection, MicroSource, USA).

### Plasmids

GFP-hPromB8LUC codes for the GFP protein under the control of the CMV promoter, and the firefly luciferase under the control of the human HSPB8 gene promoter. This plasmid was obtained by excising the promB8LUC sequence from the promB8 plasmid^13^ using KpnI-HpaI sites and, after blunting ends, cloning it into the AflII cutting-site of the pEGFP-N1 vector (Clontech Lab; Mountain View, CA, USA). pRL-TK was from Promega (Madison, WI, USA). pcDNA3 was obtained from Life Technologies Corporation (Carlsbad, CA, USA). The pEGFP-TDP-43 and pEGFP-TDP-25 code for the GFP-fused TDP-43 full-length protein (GFP-TDP-43) and its C-terminal 25 KDa (GFP-TDP-25) fragment, respectively, and were kindly provided from Dr. Petrucelli^79^. pCI-HSPB8 plasmid codes for human HSPB8^45^.

### Cell culture, transfection and treatments

NSC34 immortalized mouse motoneuronal cell line^80^,^81^ is routinely cultured and maintained in our lab^82^,^83^,^84^,^85^. NSC34 cells were plated at (i) 3.5*10^4^ cells/well in 24-well multiwell plate with coverslips for immunofluorescence analysis; (ii) 4*10^4^ cells/well in 24-well multiwell plate for transcriptional activity assay; (iii) 8*10^4^ cells/well in 12-well multiwell plate for Western Blot (WB) and Filter Retardation Assay (FRA) analyses. 24 hrs after plating, cells were transiently transfected with Lipofectamine (Life Technologies Corporation; Carlsbad, CA, USA)/transferrin (Sigma-Aldrich), following Manufacture’s instructions, as described in^13^.

Stably transfected NSC34-GFP-hPromB8LUC cells were obtained as described in^86^, by transiently transfecting NSC34 cells with the GFP-hPromB8LUC plasmid and selecting positive clones (#Ns) with G418 (400 μg/ml) for 3 weeks. Selected clones were cultured in high-gluocose DMEM supplemented with 5% FBS (Sigma-Aldrich) and G418 (100 μg/ml), and were plated at 4*10^4^ cells/well in 24-well multiwell plates, for transcriptional activity assay.

SH-SY5Y human neuroblastoma cell line was cultured in DMEM/F12 supplemented with 10% FBS (Sigma-Aldrich), Pen/Strep (5 U/mL of penicillin and 5 μg/mL of streptomycin), glutamine (2.5 mM). SH-SY5Y cells were plated at (i) 2*10^5^ cells/well in 12-well multiwell plate for Q-PCR, WB and FRA analyses; (ii) 1.5*10^4^ cells/well in 24-well multiwell plate with coverslips for immunofluorescence analysis. Cells were transiently transfected 24 hrs after plating with Lipofectamine 2000 (Life Technologies), using i) GFP-TDP-43 or GFP-TDP-25 plasmids (1 μg) and Lipofectamine (4 μl; amounts for 1 well of a 12-well multiwell); ii) ON-TARGETplus TFEB siRNA or scramble siRNA (40 pmol) (Dharmacon, Thermo Scientific Life Sciences Research, Waltham, MA, USA) and Lipofectamine (2 μl; amounts for 1 well of a 12-well multiwell), following manufacturer’s instructions. Where indicated, cells were treated with different compounds for the last 24 hrs prior to extraction. For heat shock experiment, cells were plated at 2*10^5^ cells/well in 12-well multiwell and, 48 hrs after plating, cells were grown for 1 hr at 42 °C and extracted or allowed to recover for 3 hrs at 37 °C prior to extraction.

### Microscopy and Immunofluorescence

NSC34 (3.5*10^4^ cells/well) or SH-SY5Y (7.5*10^4^ cells/well) cells plated in 24-well multiwell plates were transfected with GFP-TDP-43 or GFP-TDP-25 plasmids (0.5 μg) and treated as indicated for 24 hrs prior to extraction. 48 hrs after transfection NSC34 transfected cells were fixed and processed as previously described^85^. The following antibodies were used: (a) rabbit polyclonal anti-P62/SQSTM1 (ab91526, Abcam, Cambridge, MA, USA; dilution 1:500 in 5% nonfat milk) to detect endogenous p62/SQSTM1 protein expression; (b) rabbit polyclonal anti-LC3 (L8918, Sigma-Aldrich; dilution 1:500 in 5% nonfat milk) to detect endogenous LC3 protein expression; (c) home-made rabbit polyclonal anti-hHSPB8 (#25, dilution 1:200 in 5% nonfat milk) to detect hHSPB8. Alexa 594 anti-rabbit secondary antibody was used (A11072, Life Technologies; dilution 1:1,000 in 5% nonfat milk). DAPI or Hoechst33342 were used to stain nuclei. An Axiovert 200 microscope (Zeiss Instruments, Oberkochen, Germany), equipped with FITC/TRITC/DAPI filters and combined with a Photometric Cool-Snap CCD camera (Roper Scientific, Trenton, NJ, USA) was used to acquire images (resolution: 63X). The Metamorph software (Universal Imaging, Downingtown, PA, USA) was used for images processing.

### Filter Retardation and Western Blot assays, Preparation of Protein Extracts and Antibodies

NSC34 cells plated at 8*10^4^ cells/well in 12-well multiwell plates were transfected with GFP-TDP-43 or GFP-TDP-25 plasmids (0.5 μg) and pCDNA3 or hHSPB8 plasmids (0.6 μg). SH-SY5Y were plated and transfected as described above. NSC34 and SH-SY5Y transfected cells were harvested 48 hrs after transfection and centrifuged 5 min at 100 g at 4 °C. The pellets were resuspended in PBS added with protease inhibitor and homogenized using slight sonication as previously described^85^,^86^. Total protein level was determined with the bicinchoninic acid method (BCA assay).

For FRA, protein extracts (1.5 μg, for NSC34 transfected cells; 9 μg for SH-SY5Y transfected cells) were loaded on 0.2 μm cellulose acetate membrane (Amersham, GE Healthcare Buckinghamshire, UK) and filtered through a Bio-Dot SF Microfiltration Apparatus (Bio-Rad Laboratories, Hercules, CA, USA).

WB analysis was performed on 12% SDS polyacrylamide gel loading 15 μg of proteins for NSC34 cells and 30 μg of proteins for SH-SY5Y cells. Samples were electro-transferred to nitrocellulose membranes (Bio-Rad) using TransBlot Turbo Apparatus (Bio-Rad). Nitrocellulose and acetate membranes were processed as previously described^85^,^86^. The following primary antibodies were used: (a) rabbit polyclonal anti-GFP HRP conjugate to detect GFP-TDP-43 and GFP-TDP-25 species (MB-0712; Vector Laboratories, Burlingame, CA, USA; dilution 1:5,000); (b) home-made rabbit polyclonal anti-HSPB8 (#25; dilution 1:1,000) to detect HSPB8; (c) mouse monoclonal anti-α-tubulin (T6199, Sigma-Aldrich; dilution 1:4,000) to detect tubulin; (d) rabbit polyclonal anti-GAPDH to detect GAPDH (sc25778; Santa Cruz Biotechnology; dilution 1:1000); (e) rabbit polyclonal anti-TFEB to detect TFEB (A303-673A; Bethyl Laboratories, Montgomery, TX, USA; dilution 1:2,500). The secondary peroxidase-conjugated antibodies were: goat anti-rabbit (sc-2004; Santa Cruz Biotechnology; dilution 1:20,000); goat anti-mouse (sc-2005, Santa Cruz Biotechnology; dilution 1:20,000). Signals were detected using Clarity™ Western ECL Blotting Substrate (Bio-Rad). 20 min room temperature stripping reaction (StripABlot, EuroClone) allowed subsequently processing of nitrocellulose membranes with different antibodies.

### Transcriptional activity assay

NSC34 cells plated at 4*10^4^ cells/well in 24-well multiwell plates were transfected with GFP-hPromB8LUC plasmid (0.25 μg) and pRL-TK plasmid (0.25 μg; used for inducible firefly luciferase activities normalization). Selected clones of stably transfected NSC34-GFP-hPromB8LUC cells (#N1 - #N10) were plated at 4*10^4^ cells/well in 24-well multiwell plates and allowed to growth for 48 hrs. Where indicated, cells were treated with MG132 (10 μM) for the last 16 hrs prior to transcriptional activity assay. Transcriptional activity was measured using the LucLite Kit (Perkin Elmer, Waltham, MA, USA) and the Dual Luciferase Assay System (Promega), according to the manufacturer’s protocols, as previously described in^13^ supplementary data. Six independent replicates were analysed for each conditions tested.

### High Throughput Screening (HTS) assay and hits validation

The #N4 clone of stably transfected NSC34-GFP-hPromB8LUC cells has been selected and used to carry out the HTS experiments. The primary screening campaign, performed in duplicate, has been conducted using the SPECTRUM Collection (MicroSource, USA) library of compounds, containing 60% of clinically used drugs, 25% of natural products and 15% of other bioactive small molecules. All dispensation steps were performed by the 96 channels pipetting head of a TECAN EVO 200 (TECAN) and compounds were added at the final concentration of 1 μM. HTS assay has been optimized by seeding 4*10^4^ cells/well in View-96-well plates (PerkinElmer), by treating them with MG132 (5 μM), used as transcriptional activator of HSPB8^13^ and technical positive control in the assay. 24 hrs later, the GFP signal was detected using the Operetta HTS imaging system (PerkinElmer) at 10x magnification with 3 fields of view/well, and subsequently the relative luminescence units were quantified with an Infinite M200 plate reader (TECAN) after dispensing 1:1 ONE-Glow substrate (Promega) and incubation for 30 min at room temperature in the dark. Images were analyzed with Columbus 2.1 (PerkinElmer) and the ratio between the luminescence units and GFP mean fluorescence intensity of the cells has been considered the read-out of the assay. Z-factor and CV, and Z-score values, calculated as already described^87^ were the parameters for assay quality and compounds ranking, respectively. Counter-screening experiments have been performed using a range of 3 doses (0.1, 1, and 10 μM) of selected compounds and by measuring the viability of #N4 cells with Alamar blue assay (Life Technology). Statistical significance was calculated by means of 2-way ANOVA and Bonferroni post-test compared with DMSO values for each drug dose. Imaging of SH-SY5Y transiently transfected with GFP-TDP-43 plasmids has been performed with Operetta instrument (Perkin Elmer) with a 20x objective; analysis of number and size (range from 1 to 10 μm^2^) of spots in GFP-positive cell population was carried out with Harmony software (Perkin Elmer).

### RNA extraction and Q-PCR analysis

SH-SY5Y cells were plated at 2*10^4^ cells/well in 12-well multiwell plate, allowed to growth for 24 hrs, and then treated for 24 hrs with a range of 3 doses of different compounds (100 nM, 500 nM, or 1 μM; as indicated), or with DMSO (negative control) or MG132 (10 μM; positive control). Treated cells were harvested and centrifuged 5 min at 100 × g at 4 °C; the pellets were resuspended in 300 μL of TRI Reagent (Sigma-Aldrich); total RNA was isolated according to manufacturer’s instructions, quantified and reverse transcribed into cDNA as previously described^11^, using the High-Capacity cDNA Archive Kit (Life Technologies) according to the manufacturer’s protocol. All primers for Q-PCR were designed using the program Primer 3 and synthesized by MWG Biotech (Ebersberg, Germany) with the following sequence: hBAG1: 5′-TTT AGA GCA GCC CGA GTG AT-3′ (forward), 5′- GAC AGC AGA CAG CCA ACA AA-3′ (reverse); hBAG3: 5′- GGG TGG AGG CAA AAC ACT AA-3′ (forward), 5′-AGA CAG TGC ACA ACC ACA GC-3′ (reverse); hHSF1: 5′- CAT GAA GCA TGA GAA TGA GGC T-3′ (forward), 5′-ACT GCA CCA GTG AGA TCA GGA-3′ (reverse); hHSPB8: 5′- AGA GGA GTT GAT GGT GAA GAC C-3′ (forward), 5′-CTG CAG GAA GCT GGA TTT TC-3′ (reverse); hMAP-LC3b: 5′-CAG CAT CCA ACC CAA AAT CCC (forward), 5′ -GTT GAC ATG GTC AGG TAC AAG-3′ (reverse); hRplP0: 5′-GTG GGA GCA GAC AAT GTG GG-3′ (forward), 5′ -TGC GCA TCA TGG TGT TCT TG-3′ (reverse); hp62/SQSTM1: 5′-CCA GAG AGT TCC AGC ACA GA-3′ (forward), 5′ -CCG ACT CCA TCT GTT CCT CA-3′ (reverse); hTFEB: 5′-GCT GAT CCC CAA GGC CAA T-3′ (forward), 5′ -TCT CCA GCT CCC TGG ACT TT-3′ (reverse). Q-PCR was performed using the iTaq SYBR Green Supermix (Bio-Rad), in a 10 μl total volume, with 500 nM primers. CFX 96 Real-Time System (Bio-Rad) was used with the following cycling conditions: 94 °C for 10 min, 40 cycles at 94 °C for 15 s and 60 °C for 1 min. Data were analysed and expressed as previously described^11^,^22^. Values were normalized to those of RplP0. All statistics were performed with ∆C_t_ values. Each experiment was carried out with four independent samples.

### Statistical analysis

Statistical analysis has been performed using Student’s t-test or 2-way ANOVA using the PRISM software (GraphPad Software, La Jolla, CA, USA).

## Additional Information

**How to cite this article**: Crippa, V. *et al.* Transcriptional induction of the heat shock protein B8 mediates the clearance of misfolded proteins responsible for motor neuron diseases. *Sci. Rep.* **6**, 22827; doi: 10.1038/srep22827 (2016).

## Supplementary Material

Supplementary Information
