# Supplementary material for: Transcriptional induction of the heat shock protein B8 mediates the clearance of misfolded proteins responsible for motor neuron diseases
Source: Sci Rep. 2016 Mar 10;6:22827. doi: 10.1038/srep22827 (PMC4785366; doi:10.1038/srep22827)
Supplement: Supplementary Information [file srep22827-s1.pdf]

Transcriptional induction of the heat shock protein B8 mediates the clearance of misfolded proteins responsible for motor neuron diseases.

Valeria Crippa, Vito G. D'Agostino, Riccardo Cristofani, Paola Rusmini, Maria E. Cicardi, Elio Messi, Rosa Loffredo, Michael Pancher, Margherita Piccolella, Mariarita Galbiati, Marco Meroni, Cristina Cereda, Serena Carra, Alessandro Provenzani, Angelo Poletti

SUPPLEMENTARY INFORMATIONS

Supplementary Table 1. List of primary screening compounds from the highest value of Z score obtained.	
**Z score**	**Compound**
21,99941565	DERRUBONE
15,20902561	OXYTETRACYCLINE
14,84720775	CYPROTERONE ACETATE
14,65913419	MEGESTROL ACETATE
13,40140129	CHLOROQUINE DIPHOSPHATE
12,92332858	CRESOL
12,51162946	KHAYASIN
12,4587408	ESTRIOL
11,65850285	DOXORUBICIN
11,55293956	AUROTHIOGLUCOSE
11,33063479	BUSSEIN
9,982033556	CEDRELONE
9,577449431	COLCHICINE
9,533782481	ESTRADIOL VALERATE
9,457701464	FLUMETHAZONE PIVALATE
9,048836736	DEXAMETHASONE
8,984789171	NITROFURANTOIN
8,783587187	SPIRONOLACTONE
8,60026128	THIABENDAZOLE
8,520780029	PROMETHAZINE HYDROCHLORIDE
8,363318295	THIMEROSAL
8,305410741	COLISTIMETHATE SODIUM
7,917647838	PRIMAQUINE DIPHOSPHATE
7,784452876	ERGONOVINE MALEATE
7,62457092	CORTISONE ACETATE
7,160143379	MELPHALAN
7,0808647	METRONIDAZOLE
6,907619389	CHLORCYCLIZINE HYDROCHLORIDE
6,645654217	GUAIFENESIN
6,613422827	EQUILIN
6,460375731	ESTRONE
6,398278097	HISTAMINE DIHYDROCHLORIDE
6,380952751	NAPHAZOLINE HYDROCHLORIDE
6,21624689	PROMAZINE HYDROCHLORIDE
6,191848845	DOPAMINE HYDROCHLORIDE
6,095710019	NORGESTREL
5,959301008	CYCLOPENTOLATE HYDROCHLORIDE
5,932436211	METHOCARBAMOL
5,92852839	ESTRADIOL
5,817663356	NOVOBIOCIN SODIUM
5,719952947	FLURANDRENOLIDE
5,673409119	MICONAZOLE NITRATE
5,552334082	DYCLONINE HYDROCHLORIDE
5,528244092	OLEANDOMYCIN PHOSPHATE
5,45525056	MERCAPTOPURINE
5,369846248	TOTARALOLAL
5,270962615	DEACETOXY-7-OXOGEDUNIN
5,227463713	HALCINONIDE
5,220591674	NITROFURAZONE
5,18928383	CHLOROTHIAZIDE

5,089974325	THEOPHYLLINE
5,060561575	CYCLIZINE
4,917026146	QUINIDINE GLUCONATE
4,914285865	6-HYDROXYANGOLENSIC ACID METHYL ESTER
4,837616744	PRAZOSIN HYDROCHLORIDE
4,754426159	HEPTAMINOL HYDROCHLORIDE
4,742038413	HYDROCORTISONE
4,731476501	METHYLPREDNISOLONE
4,678991514	CYTARABINE
4,646633652	NOREPINEPHRINE
4,623513194	LIPOAMIDE
4,615838811	FLUOCINONIDE
4,602055626	ETHAMBUTOL HYDROCHLORIDE
4,548407975	TRIAMCINOLONE
4,524778254	PHENTOLAMINE HYDROCHLORIDE
4,51716078	1,3-DIDEACETYL-7-DEACETOXY-7-OXOKHIVORIN
4,387834766	MOXALACTAM DISODIUM
4,272578138	BIOCHANIN A
4,213161243	PACLITAXEL
4,177256109	DESOXYCORTICOSTERONE ACETATE
4,155330201	METAPROTERENOL
4,130393957	CLOXYQUIN
4,129104007	CAFESTOL
4,125440515	MESTRANOL
4,042779823	ETHACRYNIC ACID
3,992468555	ADENOSINE PHOSPHATE
3,956954257	EPIRUBICIN HYDROCHLORIDE
3,938537157	NORETHYNODREL
3,930979975	PROCHLORPERAZINE EDISYLATE
3,918400805	BETAMETHASONE VALERATE
3,870344581	5-FLUOROINDOLE-2-CARBOXYLIC ACID
3,852622425	EUGENYL BENZOATE
3,851890545	STREPTOMYCIN SULFATE
3,813996618	DAPSONE
3,807143404	ORPHENADRINE CITRATE
3,792294757	QUINIC ACID
3,714438406	HYDROQUINIDINE
3,713209871	HYDROCORTISONE HEMISUCCINATE
3,709652796	ERGOCALCIFEROL
3,617559664	ADENINE
3,600675526	LIDOCAINE HYDROCHLORIDE
3,5926021	alpha-DIHYDROGEDUNOL
3,542981615	SOLANESOL
3,538792792	SOLIDAGENONE
3,516913525	PODOTOTARIN
3,508416435	CHLORAMPHENICOL PALMITATE
3,450898421	PREDNISOLONE ACETATE
3,436374164	DICYCLOHEXYLUREA
3,426925607	SALICYL ALCOHOL
3,416672618	ETHINYL ESTRADIOL
3,395741129	ANDIROBIN
3,382117241	DACARBAZINE

3,373444473	CYCLOPHOSPHAMIDE HYDRATE
3,360617687	N,N-HEXAMETHYLENEAMILORIDE
3,357189629	THIODIGLYCOL
3,318520275	BUTAMBEN
3,309720013	CYCLOSERINE
3,279551744	FENBENDAZOLE
3,272186697	CEPHAPIRIN SODIUM
3,257527326	MEBENDAZOLE
3,25533315	VIDARABINE
3,234759559	EMETINE
3,233369755	DEMECLOCYCLINE HYDROCHLORIDE
3,208484808	FLUOCINOLONE ACETONIDE
3,207922299	TRISODIUM ETHYLENEDIAMINE TETRACETATE
3,16205863	SALICYLAMIDE
3,155586309	PYRANTEL PAMOATE
3,144504698	DEXAMETHASONE ACETATE
3,134489425	MITOXANTHRONE HYDROCHLORIDE
3,126921382	DYPHYLLINE
3,068252029	CROMOLYN SODIUM
3,057242681	PROPYLTHIOURACIL
3,055228284	EUGENOL
2,971531425	CEVADINE
2,958947143	1,7-DIDEACETOXY-1,7-DIOXO-3-DEACETYLKHIVORIN
2,956547738	TOMATIDINE HYDROCHLORIDE
2,952286971	ACONITINE
2,948725804	DEXAMETHASONE SODIUM PHOSPHATE
2,945503721	METHOXSALEN
2,931509589	AZELAIC ACID
2,930756131	SULFISOXAZOLE
2,928825955	4-O-METHYLPHLORACETOPHENONE
2,918712667	SODIUM OXYBATE
2,91357881	DEFEROXAMINE MESYLATE
2,913074373	KHAYANTHONE
2,87076817	ALTHIAZIDE
2,864370274	alpha-CYANO-4-HYDROXYCINNAMIC ACID
2,849767868	DEOXYGEDUNIN
2,799138039	3,7-DIMETHOXYFLAVONE
2,797389759	OXYPHENBUTAZONE
2,796988759	PROCAINE HYDROCHLORIDE
2,720560304	NADIDE
2,714381393	XYLOMETAZOLINE HYDROCHLORIDE
2,711526373	CHLORAMPHENICOL
2,688291892	CAPERATIC ACID
2,683949624	CONIFERYL ALCOHOL
2,681959376	FLUOROURACIL
2,681392813	DEOXYANDIROBIN LACTONE
2,68107157	METHYL EVERNINATE
2,674762284	IVERMECTIN
2,674611651	ORSELLINIC ACID
2,65567141	DESIPRAMINE HYDROCHLORIDE
2,644186389	CANRENOIC ACID, POTASSIUM SALT
2,64276075	SODIUM SALICYLATE

2,637299255	1,2alpha-EPOXYDEACETOXYDIHYDROGEDUNIN
2,627414361	METHYLMETHANE SULFONATE
2,604466348	LINDANE
2,597256395	beta-DIHYDROGEDUNOL
2,593881103	PYRVINIUM PAMOATE
2,589595745	CHLORTETRACYCLINE HYDROCHLORIDE
2,588708291	CLINDAMYCIN PALMITATE HYDROCHLORIDE
2,569361344	DIFLUBENZURON
2,56741524	HETEROPEUCENIN, METHYL ETHER
2,558398015	3-DEOXO-3beta-ACETOXYDEOXYDIHYDROGEDUNIN
2,545685734	DISOPYRAMIDE PHOSPHATE
2,526298738	JUAREZIC ACID
2,518506461	INDOLE-2-CARBOXYLIC ACID
2,513901691	SMILAGENIN ACETATE
2,507905251	SPHONDIN
2,507410078	ESTRADIOL CYPIONATE
2,504520645	BECLOMETHASONE DIPROPIONATE
2,504247309	FLUDROCORTISONE ACETATE
2,494237016	MEDRYSONE
2,489325852	APIOLE
2,473089931	AMINACRINE
2,46964381	GUANETHIDINE SULFATE
2,468717851	SALICYLANILIDE
2,457746504	DANAZOL
2,452924919	SULFADIAZINE
2,44985331	HYDROCORTISONE PHOSPHATE TRIETHYLAMINE
2,449565281	HYDRALAZINE HYDROCHLORIDE
2,434713933	HYDROXYZINE PAMOATE
2,428700194	SULFACETAMIDE
2,420324069	FLURBIPROFEN
2,416116463	METHYLTHIOURACIL
2,412816672	STREPTOZOSIN
2,407317009	HEXYLRESORCINOL
2,398880917	THEAFLAVIN MONOGALLATES
2,393904177	MAFENIDE HYDROCHLORIDE
2,37897749	DIPHENYLPYRALINE HYDROCHLORIDE
2,369623875	NITROMIDE
2,365499349	SISOMICIN SULFATE
2,356864663	TRIFLUMURON
2,338413268	METHIMAZOLE
2,325994266	TRYPTAMINE
2,322457031	CAPTOPRIL
2,321604851	TETRACAINE HYDROCHLORIDE
2,320517858	ETHIONAMIDE
2,313487375	SENNOSIDE B
2,298394236	TROPINE
2,285477439	RIFAMPIN
2,284348237	DEXTROMETHORPHAN HYDROBROMIDE
2,271358405	DIBUTYL PHTHALATE
2,265359798	FLUNISOLIDE
2,26109939	ISONIAZID
2,259806708	CHLORAMBUCIL

2,25515977	DEOXYSAPPANONE B 7,4'-DIMETHYL ETHER
2,254016105	DEOXYKHIVORIN
2,251711305	HYDROCHLOROTHIAZIDE
2,241520556	FUROSEMIDE
2,237150472	DISULFIRAM
2,229434985	ZOXAZOLAMINE
2,201076871	ETHIONINE
2,19708117	SULFAPYRIDINE
2,191572687	GENISTEIN
2,181238786	alpha-HYDROXYDEOXYCHOLIC ACID
2,167598502	INDOLE-3-CARBINOL
2,165311525	ETHACRIDINE LACTATE
2,150875721	THIOPENTAL SODIUM
2,149631556	ISOPROTERENOL HYDROCHLORIDE
2,14584355	METHOTREXATE(+/-)
2,140287474	SULFAMETHAZINE
2,135895021	SPECTINOMYCIN HYDROCHLORIDE
2,131214619	RESVERATROL 4'-METHYL ETHER
2,1257765	ERYTHROMYCIN ETHYLSUCCINATE
2,119995368	ETHOPROPAZINE HYDROCHLORIDE
2,118184234	DIALLYL SULFIDE
2,10910262	DIOSMETIN
2,107926186	METHYL DEOXYCHOLATE
2,104911051	CHLORPHENIRAMINE (S) MALEATE
2,101947859	ALBENDAZOLE
2,094655897	ERYTHROMYCIN
2,086626054	ISOFLUPREDNONE ACETATE
2,078448047	PREDNISOLONE
2,078448047	PROGESTERONE
2,078448047	PYRIMETHAMINE
2,07532281	DIPHENHYDRAMINE HYDROCHLORIDE
2,075100431	SULINDAC
2,069250367	PROTOPORPHYRIN IX
2,067200264	RUTOSIDE (rutin)
2,06338364	DEHYDROVARIABILIN
2,061711501	BOLDINE
2,057528558	QUINACRINE HYDROCHLORIDE
2,044779159	HYDROXYUREA
2,035040942	2,4-DICHLOROPHENOXYACETIC ACID, ISOOCTYL ESTER
2,015128284	KINETIN
2,004770644	AMINOPYRINE
2,001180239	TESTOSTERONE PROPIONATE
2,00110743	HALOPERIDOL
1,996194122	2-HYDROXY-5 (6)EPOXY-TETRAHYDROCARYOPHYLLENE
1,99052752	PUTRESCINE DIHYDROCHLORIDE
1,983769744	COENZYME B12
1,979663792	RETUSIN 7-METHYL ETHER
1,975745203	RESORCINOL
1,974880117	CURCUMIN
1,972314109	RHODINYL ACETATE
1,972095109	beta-AMYRIN ACETATE
1,958576555	SULFAMETHIZOLE

1,958455129	MITOTANE
1,944207407	3-PINANONE OXIME
1,939880069	BENSERAZIDE HYDROCHLORIDE
1,924679636	DEOXYGEDUNOL ACETATE
1,917487625	BETAMETHASONE
1,894935802	ROSOLIC ACID
1,894057777	CHLOROXYLENOL
1,889710163	NIMESULIDE
1,882484804	HELENINE
1,877499249	DANTHRON
1,873400637	ASPIRIN
1,867403454	ALDRIN
1,864041871	4'-METHOXYFLAVONE
1,861075508	KUHLMANNIN
1,857605358	XYLOCARPUS A
1,855673968	HETACILLIN POTASSIUM
1,848142521	8beta-HYDROXYCARAPIN, 3,8-HEMIACETAL
1,848004075	METAXALONE
1,846821627	RACEPHEDRINE HYDROCHLORIDE
1,844981235	MEPARTRICIN
1,843252895	PYRITHIONE ZINC
1,836913198	3,16-DIDEOXYMEXICANOLIDE-3beta-DIOL
1,834505141	3-AMINO-1,2,4-TRIAZOLE
1,823225307	TOLAZOLINE HYDROCHLORIDE
1,804289728	METHYL ORSELLINATE
1,798041558	MUCIC ACID
1,796681279	NORHARMAN
1,787598251	CORALYNE CHLORIDE
1,773645668	CARMOFUR
1,763726734	OXOTREMORINE SESQUIFUMARATE
1,763395798	HOMATROPINE METHYLBROMIDE
1,754009656	2-METHOXYRESORCINOL
1,754003845	MELIBIOSE
1,750908426	EVERNINIC ACID
1,74752469	ESTRAGOLE
1,73698137	HYDROCORTISONE ACETATE
1,724182299	HEXACHLOROPHENE
1,716261999	CLOBETASOL PROPIONATE
1,705504946	8-HYDROXYCARAPINIC ACID
1,704130632	3,4-DIHYDROXYCARANE
1,698418517	PROCYCLIDINE HYDROCHLORIDE
1,693115934	TOTAROL ACETATE
1,681346033	GUANABENZ ACETATE
1,679679561	SUXIBUZONE
1,679676368	ALIZARIN
1,676720977	TERBUTALINE HEMISULFATE
1,668100527	UTILIN
1,65657366	7-NITROINDAZOLE
1,650170998	DEOXYSAPPANONE B 7,3'-DIMETHYL ETHER ACETATE
1,648183776	KHIVORIN
1,646041563	BENTAZON
1,643594193	NIFENAZONE

1,635011103	PROBUCOL
1,629936267	DICLOFENAC SODIUM
1,628679613	ACETAMINOPHEN
1,623779292	NICLOSAMIDE
1,613504227	AURAPTENE
1,597050982	VALPROATE SODIUM
1,596578727	CHRYSIN DIMETHYL ETHER
1,596071214	1,4,5,8-TETRAHYDROXY-2,6-DIMETHYLANTHROQUINONE
1,591611509	CIANIDANOL
1,590116475	HESPERETIN
1,590063904	TAMOXIFEN CITRATE
1,582106584	7-DESACETOXY-6,7-DEHYDROGEDUNIN
1,578357323	HYGROMYCIN B
1,571054449	DIFFRACTAIC ACID
1,570884614	d,l-threo-3-HYDROXYASPARTIC ACID
1,567057282	PSEUDO-ANISATIN
1,566159136	DOXEPIN HYDROCHLORIDE
1,564021756	ACETAZOLAMIDE
1,560856175	KETOTIFEN FUMARATE
1,557726557	DESOXYMETASONE
1,553564477	GRAMICIDIN
1,552996105	EPICATECHIN
1,552582378	beta-AMYRIN
1,53828471	3-DESHYDROXYSAPPANOL TRIMETHYL ETHER
1,52412461	PICROTIN
1,518202248	CEPHARANTHINE
1,515739837	LITHOCHOLIC ACID
1,51163494	SULFAMETHOXAZOLE
1,505629949	HEDERACOSIDE C
1,502645913	LATHOSTEROL
1,494370165	EPOXY (4,5alpha)-4,5-DIHYDROSANTONIN
1,49422758	RAMIFENAZONE
1,492989341	SINOMENINE
1,491111816	URSODIOL
1,48994271	CHOLIC ACID, METHYL ESTER
1,485611447	DIURON
1,48364971	3beta-HYDROXYISOALLOSPIROST-9(11)-ENE
1,483284075	3-NOR-3-OXOPANASINSAN-6-OL
1,482256159	METHSCOPOLAMINE BROMIDE
1,481276866	CHOLESTEROL
1,476698178	HYDROFLUMETHIAZIDE
1,475284253	SCOPOLAMINE HYDROBROMIDE
1,473433392	3-HYDROXYFLAVONE
1,471502265	PHENAZOPYRIDINE HYDROCHLORIDE
1,467445266	AMCINONIDE
1,464367813	HAEMATOPORPHYRIN
1,454316048	CARBIMAZOLE
1,453132239	CARAPIN-8(9)-ENE
1,448429237	TANGERITIN
1,447502122	BETULINIC ACID
1,443013713	FARNESOL
1,433152811	DIHYDRODEOXYGEDUNIN

1,43210957	LEVONORDEFRIN
1,424891538	SWIETENOLIDE DIACETATE
1,417801391	ACADESINE
1,409767012	MECLOFENOXATE HYDROCHLORIDE
1,408320676	ENOXOLONE
1,405010004	DOXYLAMINE SUCCINATE
1,397860302	MEXICANOLIDE
1,39689943	BENZO[a]PYRENE
1,383251112	BICUCULLINE (+)
1,382693792	3-DEOXO-3beta-HYDROXYMEXICANOLIDE 16-ENOL ETHER
1,382611724	HECOGENIN ACETATE
1,378995715	GALLAMINE TRIETHIODIDE
1,37211732	BENZTROPINE
1,370552722	2,4-DICHLOROPHENOXYACETIC ACID, METHYL ESTER
1,364724279	MANNITOL
1,361441906	NORTRIPTYLINE
1,357631598	ETHYL PARABEN
1,354496986	NEOMYCIN SULFATE
1,354076182	IPRATROPIUM BROMIDE
1,352267083	CEFADROXIL
1,351349975	BACLOFEN
1,342707561	BISACODYL
1,341695647	FUSIDIC ACID
1,340546801	PYRITHYLDIONE
1,339456647	FORMONONETIN
1,33707305	3,4-DIMETHOXYDALBERGIONE
1,333976757	DESONIDE
1,329529275	TODRALAZINE HYDROCHLORIDE
1,325314357	2,5-DIHYDROXY-3,4-DIMETHOXY-4'-ETHOXYBENZOPHENONE
1,32481936	BENZOCAINE
1,322083285	ATOMOXETINE HYDROCHLORIDE
1,317457774	DOCOSANOL
1,313957514	AVOCADYNE ACETATE
1,309780604	LAPACHOL
1,30811264	PROMETON
1,300858413	HALAZONE
1,300690279	QUININE SULFATE
1,298535436	PRAZIQUANTEL
1,298213987	PICROTOXININ
1,296561534	LUPANINE PERCHLORATE
1,295997739	alpha-MANGOSTIN
1,295020049	DILTIAZEM HYDROCHLORIDE
1,285883852	CULMORIN
1,285608738	QUININE ETHYL CARBONATE
1,278730524	DEXPROPRANOLOL HYDROCHLORIDE
1,278055519	PURPUROGALLIN
1,276749234	TRIMEDLURE
1,269959584	DERRUSTONE
1,259803594	TRAMADOL HYDROCHLORIDE
1,255128749	BETAMETHAZONE SODIUM PHOSPHATE
1,255062907	HOMATROPINE BROMIDE
1,253729756	GLUTAMINE (L)

1,253419952	LUFENURON
1,252635756	CHLOROPHYLL
1,251687956	SULFABENZAMIDE
1,250080181	THEANINE
1,249465357	TYRAMINE
1,247068828	RHIZOCARPIC ACID
1,242745216	PROCAINAMIDE HYDROCHLORIDE
1,237836694	ADENOSINE
1,237293038	DIMETHOATE
1,230466728	4-NAPHTHALIMIDOBUTYRIC ACID
1,227545988	ANTHRAQUINONE
1,2262099	AMINOSALICYLATE SODIUM
1,221275354	CONVALLATOXIN
1,218952905	VINBLASTINE SULFATE
1,218932715	GLYCYRRHIZIC ACID, AMMONIUM SALT
1,215313487	1R,9S-HYDRASTINE
1,212822831	3-METHOXYCATECHOL
1,208754803	THIRAM
1,208220072	PROMETRYN
1,207266811	FURAZOLIDONE
1,20474908	PYRROMYCIN
1,202245765	HYDRASTINE (1R, 9S)
1,199322064	BUDESONIDE
1,19860674	PYRILAMINE MALEATE
1,197964402	CATECHIN TETRAMETHYLETHER
1,196956046	VALERYL SALYCILATE
1,196320424	4-AMINOETHYLBENZENESULFONYL FLUORIDE HYDROCHLORIDE
1,193059128	HYDROCORTISONE VALERATE
1,193057317	CHAULMOOGRIC ACID
1,192962776	OCTODRINE
1,191155886	PRIMIDONE
1,190624131	8-HYDROXY-15,16-BISNOR-11-LABDEN-13-ONE
1,188573413	PHENETHICILLIN POTASSIUM
1,184860208	IMIPRAMINE HYDROCHLORIDE
1,184404422	DULOXETINE HYDROCHLORIDE
1,182839694	MEDROXYPROGESTERONE ACETATE
1,18191069	AZATHIOPRINE
1,180680097	EPICHOLESTANOL
1,18061382	AMINOETHOXYDIPHENYLBORANE
1,177290014	1,4-NAPHTHOQUINONE
1,177110464	METHIONYL-LEUCYLPHENYLALANINE ACETATE
1,171482859	TRIAMCINOLONE ACETONIDE
1,171379505	TEMEFOS
1,170459557	7-DEACETYLKHIVORIN
1,169918687	PURPUROGALLIN-4-CARBOXYLIC ACID
1,167552091	CHLORPROPHAM
1,166405735	ERYTHROSINE SODIUM
1,165653709	CHRYSANTHEMIC ACID, ETHYL ESTER
1,164971805	3-HYDROXY-3',4'-DIMETHOXYFLAVONE
1,163144654	MUUROLLADIE-3-ONE
1,159457492	CAPSANTHIN
1,157627732	DICHLORODIPHENYLDICHLOROETHYLENE

1,155761724	AMPICILLIN SODIUM
1,152812448	PALMATINE CHLORIDE
1,152077664	BUFEXAMAC
1,150938988	DIHYDROFISSINOLIDE
1,15088308	EPOXYGEDUNIN
1,148611491	TETRACHLOROISOPHTHALONITRILE
1,146463468	ACRISORCIN
1,144965755	KHELLIN
1,140103821	MOLSIDOMINE
1,138676209	ROXARSONE
1,13855537	AMPROLIUM
1,138405063	alpha-TOCHOPHEROL
1,137373014	CEPHALORIDINE
1,135454856	BENZTHIAZIDE
1,131978511	ANTAZOLINE PHOSPHATE
1,130847128	CHLOROGENIC ACID
1,128315116	IDEBENONE
1,124208318	DJENKOLIC ACID
1,124139784	COLFORSIN
1,123763695	TETRAHYDROZOLINE HYDROCHLORIDE
1,123186764	AZACITIDINE
1,122071955	GUANIDINE CARBONATE
1,121598292	SODIUM NITROPRUSSIDE
1,1207604	BROMOPRIDE
1,119592439	AZTREONAM
1,118807013	MADECASSIC ACID
1,118750383	ROSUVASTATIN CALCIUM
1,11712832	3,3'-DIINDOLYLMETHANE
1,113539928	IRIGINOL HEXAACEATATE
1,112289609	DEOXYSAPPANONE B 7,3'-DIMETHYL ETHER
1,108412798	TOLTERODINE TARTRATE
1,106668647	ACETYLCYSTEINE
1,105271867	PRENYLETIN
1,101596638	KINETIN RIBOSIDE
1,096704612	QUINAMIDE ISOPROPYLIDENE
1,095108562	DIOSGENIN
1,091056306	AMOXICILLIN
1,091035408	COUMARIN
1,090433776	MENTHYL BENZOATE
1,089316788	3beta-ACETOXYDEOXODIHYDROGEDUNIN
1,087685036	SALSALATE
1,080521697	SANGUINARINE SULFATE
1,078676062	DIPYRONE
1,074249839	AMITRIPTYLINE HYDROCHLORIDE
1,073830473	OXYBUTYNIN CHLORIDE
1,072173176	PENTYLENETETRAZOL
1,07089445	PODOPHYLLOTOXIN ACETATE
1,07027796	ASARININ (-)
1,06628107	ICARIIN
1,065900181	TRIMEBUTINE MALEATE
1,062802818	CINCHONIDINE
1,060517469	SPARTEINE SULFATE

1,057941685	HYDROCORTISONE BUTYRATE
1,057077592	2,4-DINITROPHENOL
1,055285135	TESTOSTERONE
1,052005753	PROPAFENONE HYDROCHLORIDE
1,051759356	BETHANECHOL CHLORIDE
1,051658635	IODOQUINOL
1,047357732	PROPANTHELINE BROMIDE
1,044072511	MERBROMIN
1,042211382	PHENACYLAMINE HYDROCHLORIDE
1,039959288	ADONITOL
1,037944085	5-HYDROXY-2',4',7,8-TETRAMETHOXYFLAVONE
1,037240664	ALLOPREGNANOLONE
1,036403763	3-HYDROXY-4-(SUCCIN-2-YL)-CARYOLANE delta-LACTONE
1,035513434	PROTOVERATRINE A
1,035429126	GRISEOFULVIN
1,035345678	LORGLUMIDE SODIUM
1,029239019	SUCRALFATE SODIUM (10% w/v in DMSO)
1,026385406	CLOVANEDIOL DIACETATE
1,025503122	alpha-METHYL-L-TYROSINE
1,025343866	AMINOHYDROXYBUTYRIC ACID
1,024359312	GARLICIN
1,022953284	RETINYL ACETATE
1,020827925	2-THIOURACIL
1,02063869	PHENYLBUTAZONE
1,019226943	ACEBUTOLOL HYDROCHLORIDE
1,017790132	MAPROTILINE HYDROCHLORIDE
1,017542793	N-METHYL-D-ASPARTIC ACID (NMDA)
1,012570151	TOLNAFTATE
1,012303252	ENDRIN
1,011779389	ANTHOTHECOL
1,010866956	GEDUNIN
1,009358636	TRIPELENNAMINE CITRATE
1,007242809	ETANIDAZOLE
1,004487621	HERNIARIN
1,001322713	3,7-EPOXYCARYOPHYLLAN-6-OL
1,000503763	L-BUTHIONINE SULFOXIMINE
0,996143386	CAFFEIC ACID
0,993629616	LANSOPRAZOLE
0,992101518	beta-CAROTENE
0,991951725	PLUMBAGIN
0,99155973	BERBAMINE HYDROCHLORIDE
0,987199195	NALIDIXIC ACID
0,986153415	FLUTAMIDE
0,982747485	ACETOHYDROXAMIC ACID
0,978569881	METOPROLOL TARTRATE
0,977744758	BERGENIN
0,966890609	3,7-DIHYDROXYFLAVONE
0,964740223	INDOMETHACIN
0,962027371	KETOPROFEN
0,959977891	PATULIN
0,95776102	VISNAGIN
0,955496099	FERULIC ACID

0,951909195	ANISINDIONE
0,951097055	3-DEOXY-3beta-HYDROXYANGOLENSIC ACID METHYL ESTER
0,948087009	2-MERCAPTOBENZOTHIAZOLE
0,946868082	HYDRINDANTIN HYDRATE
0,940622974	ESTRONE BENZOATE
0,938608712	CEDROL
0,935986917	SULFAMERAZINE
0,935487643	DAUNORUBICIN
0,935254097	2,3-DICHLORO-5,8-DIHYDROXYNAPTHOQUINONE
0,935196354	ACONITIC ACID
0,930992592	OLEANOIC ACID
0,930299793	DIHYDROMUNDULETONE
0,924489674	METHYLERGONOVINE MALEATE
0,92338921	LEVOTHYROXINE
0,923170269	NALBUPHINE HYDROCHLORIDE
0,919418938	MIMOSINE
0,91818711	BIXIN
0,917868578	CLOPAMIDE
0,91371874	BENAZEPRIL HYDROCHLORIDE
0,912298641	CYSTEAMINE HYDROCHLORIDE
0,910340244	7-DEACETOXY-7-OXOKHIVORIN
0,90859633	CLOFIBRATE
0,907842218	USNIC ACID
0,906765652	GEMFIBROZIL
0,905295904	BISSALICYL FUMARATE
0,901743963	AMODIAQUINE DIHYDROCHLORIDE
0,898979887	PREDNISOLONE HEMISUCCINATE
0,897109014	DEGUELIN(-)
0,894979448	CARBETAPENTANE CITRATE
0,894909832	PERILLYL ALCOHOL
0,889716384	RITANSERIN
0,889156857	REBAMIPIDE
0,885068202	HYDRASTININE HYDROCHLORIDE
0,884437294	HYOSCYAMINE
0,883258855	HYDROLYSIS PRODUCT OF BUSSEIN
0,882934254	AMPHOTERICIN B
0,88195869	CLOMIPHENE CITRATE
0,879490492	MINOCYCLINE HYDROCHLORIDE
0,878923682	APOTOXICAROL
0,878747207	METHOMYL
0,876470938	PIPEMIDIC ACID
0,874364961	PIPENZOLATE BROMIDE
0,873771721	FENBUFEN
0,872997651	KHAYASIN C
0,869192008	LUPININE
0,863818439	NYSTATIN
0,863754835	GUAIOL(-)
0,855499917	MELENGESTROL ACETATE
0,8544885	ANTHRALIN
0,852839878	GENTAMICIN SULFATE
0,852809908	KAINIC ACID
0,852057463	ARABITOL(D)

0,849620104	CINEOLE
0,847523087	CITRULLINE
0,845791319	TRIMETHADIONE
0,843420932	RHOIFOLIN
0,842473207	HARMALOL HYDROCHLORIDE
0,833303098	ESCULIN MONOHYDRATE
0,832947669	ALVERINE CITRATE
0,830255086	CITRININ
0,828936405	GLUTAMINE (D)
0,828607633	ACETANILIDE
0,825991393	HARMINE
0,82427338	DIPYRIDAMOLE
0,822485542	DIHYDROGEDUNIN
0,821319224	LUNARINE
0,817450155	PSEUDOEPHEDRINE HYDROCHLORIDE
0,815576018	CEDRYL ACETATE
0,815388578	SELEGILINE HYDROCHLORIDE
0,814891707	ISOXSUPRINE HYDROCHLORIDE
0,812188762	MANGIFERIN
0,810475632	ISOKOBUSONE
0,810248151	6-AMINONICOTINAMIDE
0,807431882	SYRINGIC ACID
0,804217614	CARYOPHYLLENE [t(-)]
0,804078255	NORETHINDRONE ACETATE
0,802424199	15-NORCARYOPHYLLEN-3-ONE
0,800444158	SORBITOL
0,799536944	SULFAQUINOXALINE SODIUM
0,796693018	GENETICIN
0,791035429	TRETINON
0,784743929	HAEMATOXYLIN PENTAACETATE
0,780775584	ERGOTAMINE TARTRATE
0,779946811	BETAMETHASONE ACETATE
0,777585062	ZOMEPIRAC SODIUM
0,776242654	NABUMETONE
0,775027923	ATRANORIN
0,77493091	ESTRADIOL PROPIONATE
0,771200334	IMIDAZOL-4-YLACETIC ACID SODIUM SALT
0,771004331	4-HYDROXY-6-METHYLPYRAN-2-ONE
0,767167162	TELENZEPINE HYDROCHLORIDE
0,764719976	ALLOPURINOL
0,764410684	OCTISALATE
0,762959238	DIBENZOYLMETHANE
0,762512518	MYCOPHENOLIC ACID
0,762105615	TRIADIMEFON
0,758777577	DECAMETHONIUM BROMIDE
0,758666366	APOMORPHINE HYDROCHLORIDE
0,757813144	L-PHENYLALANINOL
0,757684997	PELLETIERINE HYDROCHLORIDE
0,755897291	XANTHONE
0,755718132	FRIEDELIN
0,755163421	PRIDINOL METHANESULFONATE
0,755106308	STRYCHNINE

0,754331154	BACAMPICILLIN HYDROCHLORIDE
0,753681981	CEPHALOTAXINE
0,752313631	PHENYLPROPANOLAMINE HYDROCHLORIDE
0,751880829	PURPURIN
0,750802641	1,7-DIDEACETOXY-1,7-DIOXOKHIVORIN
0,749581939	ANISOMYCIN
0,745860466	3,7-EPOXYCARYOPHYLLAN-6-ONE
0,744480122	AMBROXOL HYDROCHLORIDE
0,742135753	2,3-DIHYDROXY-4-METHOXY-4'-ETHOXYBENZOPHENONE
0,742068834	PHYSOSTIGMINE SALICYLATE
0,741662646	CHLORANIL
0,741527996	NICARDIPINE HYDROCHLORIDE
0,74123478	METHYLDOPA
0,740900623	DIBUCAINE HYDROCHLORIDE
0,740655488	DEBRISOQUIN SULFATE
0,739587249	GOSSYPETIN
0,738131772	4'-METHOXYCHALCONE
0,737038875	URSOCHOLANIC ACID
0,736048856	CARNOSINE
0,735159205	TRAZODONE HYDROCHLORIDE
0,731577117	AMILORIDE HYDROCHLORIDE
0,726036929	YOHIMBINE HYDROCHLORIDE
0,726020718	CARMUSTINE
0,725835736	URSOLIC ACID
0,72272443	ENTANDROPHRAGMIN
0,72111209	TRIOXSALEN
0,720815451	APIGENIN
0,717846787	SULFINPYRAZONE
0,716970226	PRONETALOL HYDROCHLORIDE
0,716258701	1R,2S-PHENYLPROPYLAMINE
0,714442691	L(+/-)-ALLIIN
0,714071814	BETAMETHASONE 17,21-DIPROPIONATE
0,713372564	AZINPHOS METHYL
0,712786753	2',4-DIHYDROXY-3,4',6'-TRIMETHOXYCHALCONE
0,710938385	CARBENOXOLONE SODIUM
0,7079269	LUPANYL ACID HYDROCHLORIDE
0,707519896	TRIPTOPHENOLIDE
0,70655795	TYLOSIN TARTRATE
0,706386209	CICLOPIROX OLAMINE
0,705430835	AMINOETHYLISOTHIOUREA DIHYDROBROMIDE
0,70253257	DIFLUNISAL
0,702097801	GOSSYPOL
0,70152095	LISINOPRIL
0,700964246	GITOXIGENIN DIACETATE
0,699869032	FUCOSTANOL
0,69574374	FENOTEROL HYDROBROMIDE
0,693610579	AMIKACIN SULFATE
0,691249668	HYDROXYPROGESTERONE CAPROATE
0,690571649	CLOTRIMAZOLE
0,686756523	TETRACYCLINE HYDROCHLORIDE
0,677351054	PIPERINE
0,671302329	MANGOSTIN TRIMETHYL ETHER

0,670434514	ARTHONIOIC ACID
0,670392098	BLASTICIDIN S
0,668529995	PIPLARTINE
0,668183944	SULFATHIAZOLE
0,667819608	PISCIDIC ACID
0,667201321	3,4',5,6,7-PENTAMETHOXYFLAVONE
0,665802056	FAMCICLOVIR
0,664672922	2-AMINOGUANIDINE HEMISULFATE
0,66121131	TIGOGENIN
0,660168357	PICROPODOPHYLLOTOXIN ACETATE
0,659055882	XYLAZINE
0,656177669	METHOXYVONE
0,654675975	MEMANTINE HYDROCHLORIDE
0,653360235	PENTACHLOROPHENOL
0,653349109	BACITRACIN
0,652309681	AGMATINE SULFATE
0,650315473	DIPHENYLUREA
0,643187679	TOTAROL
0,640016183	SECURININE
0,639603964	EXALAMIDE
0,639249406	PIROXICAM
0,639021173	CUNEATIN METHYL ETHER
0,638189464	CHOLECALCIFEROL
0,637256894	DEXPANTHENOL
0,635259751	PRALIDOXIME MESYLATE
0,634326576	CHENODIOL
0,633678233	PROXYPHYLLINE
0,633612795	GITOXIN
0,632774397	ACETARSOL
0,630939556	CEPHALOTHIN SODIUM
0,628129171	PYROCATECHUIC ACID
0,625397948	RUTILANTINONE
0,625395372	FENBUTYRAMIDE
0,624090031	DESOXYPEGANINE HYDROCHLORIDE
0,624059698	FLUPHENAZINE HYDROCHLORIDE
0,623501911	METHYLDOPATE HYDROCHLORIDE
0,623077224	ACETYL-L-LEUCINE
0,623007122	EPHEDRINE (1R,2S) HYDROCHLORIDE
0,622946421	AMANTADINE HYDROCHLORIDE
0,622103874	CYCLOVERATRYLENE
0,621072755	VANCOMYCIN HYDROCHLORIDE
0,615901414	QUERCETIN
0,61571454	PHENTERMINE
0,613028457	SAPPANONE A DIMETHYL ETHER
0,61037532	CLOZAPINE
0,610207183	ALOIN
0,609684708	BEKANAMYCIN SULFATE
0,609054627	DEHYDRO (11,12)URSOLIC ACID LACTONE
0,6087896	3,4'-DIHYDROXYFLAVONE
0,608417609	PREDNISONE
0,608324525	EPIGALLOCATECHIN-3-MONOGALLATE
0,608078336	SALSOLINE

0,607237154	THIOGUANINE
0,606030282	MEFEXAMIDE
0,6048182	ANAGRELIDE HYDROCHLORIDE
0,596370171	HUPERZINE A
0,595515925	MELATONIN
0,594586038	3-ISOBUTYL-1-METHYLXANTHINE (IBMX)
0,59191076	LARIXOL ACETATE
0,591843509	LINAMARIN
0,591541971	ROFECOXIB
0,591465156	APIIN
0,591035833	COUMOPHOS
0,590727132	ACRIFLAVINIUM HYDROCHLORIDE
0,590015132	HECOGENIN
0,588745816	SOLASODINE
0,588600095	UREA
0,585807245	alpha-TOCHOPHERYL ACETATE
0,584784149	IRIDIN
0,58473942	LIOTHYRONINE
0,581989473	NAFCILLIN SODIUM
0,580678168	RESERPINE
0,580615093	CARBIDOPA
0,579540631	OBLIQUIN
0,578759136	EPIGALLOCATECHIN
0,576967201	MUNDULONE ACETATE
0,576694107	TYROTHRICIN
0,576332251	PENTOXIFYLLINE
0,572182109	SULFANITRAN
0,571575706	PROBENECID
0,571244471	RETINYL PALMITATE
0,567315477	GUVACINE HYDROCHLORIDE
0,567154128	BITHIONATE SODIUM
0,566433326	SPERMIDINE TRIHYDROCHLORIDE
0,565425196	HYDROQUINONE
0,564847794	TETROQUINONE
0,564321743	PYRITINOL
0,563869644	GENTIAN VIOLET
0,563678671	METHYLPREDNISOLONE SODIUM SUCCINATE
0,563651492	AZOBENZENE
0,561745715	HYDROCOTARNINE HYDROBROMIDE
0,561506436	7,4'-DIHYDROXYFLAVONE
0,560791686	ERYTHROSE
0,560169831	ATRAZINE
0,55897189	PHYTONADIONE
0,555941017	GINKGOLIC ACID
0,555878465	5-CHLOROINDOLE-2-CARBOXYLIC ACID
0,553866956	AKLOMIDE
0,553664167	CARBENICILLIN DISODIUM
0,553495672	AVOCADYNE
0,552353456	MECYSTEINE HYDROCHLORIDE
0,550839603	MEROGEDUNIN
0,547147883	ATROPINE SULFATE
0,546666959	p-FLUOROPHENYLALANINE

0,545362502	NORGESTIMATE
0,544230109	BISABOLOL
0,54384163	5-AMINOPENTANOIC ACID HYDROCHLORIDE
0,542958834	ALANYL-dl-LEUCINE
0,542298522	CHLOROTRIANISENE
0,541818592	TRIAMTERENE
0,536404872	OXYPHENCYCLIMINE HYDROCHLORIDE
0,534379321	DROPROPIZINE
0,534274544	DIBENZOTHIOPHENE
0,533447314	3,4-DIDESMETHYL-5-DESHYDROXY-3'-ETHOXYSCLEROIN
0,532176624	BETAHISTINE HYDROCHLORIDE
0,531749944	4,4'-DIISOTHIOCYANOSTILBENE-2,2'-SUFONIC ACID SODIUM SALT
0,53105153	FLUNARIZINE HYDROCHLORIDE
0,53038014	RHODOCLADONIC ACID
0,529443665	MENTHOL(-)
0,529151735	DEOXYCHOLIC ACID
0,527092869	ALBUTEROL (+/-)
0,52503698	LEVOFLOXACIN
0,522257376	SAPPANONE A TRIMETHYL ETHER
0,52209621	BRAZILIN
0,521136849	HYPOXANTHINE
0,517730486	CHOLIC ACID
0,517730002	CYSTAMINE DIHYDROCHLORIDE
0,517623043	POLYMYXIN B SULFATE
0,517122231	RAMIPRIL
0,516647754	GERANYLGERANIOL
0,513131291	SINENSETIN
0,51260734	SEMUSTINE
0,510847986	MINAPRINE HYDROCHLORIDE
0,510244204	DEXCHLORPHENIRAMINE MALEATE
0,509914766	PROSCILLARIDIN
0,50966769	MIGLITOL
0,508016225	CHLORHEXIDINE
0,507532682	CHROMOCARB
0,507213032	INOSITOL
0,507120854	NORFLOXACIN
0,504557121	FAMOTIDINE
0,503859358	3,4-DIMETHOXYCINNAMIC ACID
0,502510941	OXCARBAZEPINE
0,501815752	AZTREONAM
0,500632082	WARFARIN
0,499505342	DESLORATIDINE
0,499263477	MECLOCYCLINE SULFOSALICYLATE
0,499242887	ACETYLCHOLINE
0,497637517	beta-ESCIN
0,49668374	VINDOLINE
0,496299541	CINNAMIC ACID
0,49554951	CHOLINE CHLORIDE
0,494328236	MOXIFLOXACIN HYDROCHLORIDE
0,493414581	HYDROXYCHLOROQUINE SULFATE
0,492820183	CEFACLOR
0,492324483	CARAPIN

0,49227866	TROXERUTIN
0,490999007	CEFOXITIN SODIUM
0,489090371	TRIACETIN
0,488669807	ANTIPYRINE
0,488285291	SULFASALAZINE
0,485427236	HYMECROMONE METHYL ETHER
0,483819166	4'-DEMETHYLEPIPODOPHYLLOTOXIN
0,483033482	OUABAIN
0,482857062	NIMODIPINE
0,481639255	4-(3-BUTOXY-4-METHOXYBENZYL)IMIDAZOLIDIN-2-ONE
0,481471355	CIMETIDINE
0,481122731	BICUCULLINE(-) METHIODIDE
0,478749625	URIDINE TRIPHOSPHATE TRISODIUM
0,478028976	N-FORMYLMETHIONYLPHENYLALANINE
0,4765714	ANHYDROGLUCOSE
0,470599375	SINAPIC ACID METHYL ETHER
0,470493701	ESTRADIOL METHYL ETHER
0,469371771	TOLMETIN SODIUM
0,468096953	DEACETYLGEDUNIN
0,465980739	MELEZITOSE
0,464731789	GLUTATHIONE
0,464584385	BATYL ALCOHOL
0,463846121	AZADIRACHTIN
0,460145268	ALPRENOLOL
0,459960015	CLOFOCTOL
0,459311956	PHYSCION
0,458869912	DICTAMNINE
0,45883271	UVAOL
0,457843069	TRIPROLIDINE HYDROCHLORIDE
0,45548409	GLUCOSAMINE HYDROCHLORIDE
0,455302481	SOTALOL HYDROCHLORIDE
0,454515241	PHENOLPHTHALEIN
0,448839206	2,4,5-TRICHLOROPHENOXYACETIC ACID
0,447159917	CLOXACILLIN SODIUM
0,446806526	TRYPTOPHAN
0,445972808	ONONETIN
0,444993907	PENTETIC ACID
0,444687562	ASARYLALDEHYDE
0,443727535	TOTAROL-19-CARBOXYLIC ACID, METHYL ESTER
0,442560041	LANATOSIDE C
0,440796688	GINKGOLIDE A
0,440491732	ISOPROPAMIDE IODIDE
0,439931726	CHLORDANE
0,437957891	MEFENAMIC ACID
0,435268905	IBUPROFEN
0,434698776	EBSELEN
0,434461372	SILIBININ
0,432280354	GUAIAZULENE
0,432156212	ISOSORBIDE DINITRATE
0,431217456	OXYMETAZOLINE HYDROCHLORIDE
0,431195667	12a-HYDROXY-5-DEOXYDEHYDROMUNDUSERONE
0,43083963	SULFAMETHOXYPYRIDAZINE

0,430769588	CYCLOLEUCINE
0,427867929	SULFAMONOMETHOXINE
0,426167882	CARBACHOL
0,425981564	FLUCONAZOLE
0,425603197	BUPIVACAINE HYDROCHLORIDE
0,421720374	FOSFOMYCIN
0,419530544	ROBUSTIC ACID
0,419517396	LOVASTATIN
0,418660027	ACETOHEXAMIDE
0,418069015	METHYL ROBUSTONE
0,417718682	THIAMYLAL SODIUM
0,417695117	SULFADOXINE
0,416797342	GLAFENINE
0,412084969	1-PHENYLBIGUANIDE HYDROCHLORIDE
0,40818315	NIFUROXAZIDE
0,407009609	QUASSIN
0,406258872	PERSEITOL
0,406157589	ESTRADIOL ACETATE
0,405969483	ISOLIQUIRITIGENIN
0,404647384	LARIXOL
0,401470318	LIQUIRITIGENIN DIMETHYL ETHER
0,400379402	TIMOLOL MALEATE
0,398541416	SIMVASTATIN
0,398089496	SODIUM DEHYDROCHOLATE
0,397755558	ALEURETIC ACID
0,39762356	THEOBROMINE
0,397468653	METHACYCLINE HYDROCHLORIDE
0,396386397	PROTIONAMIDE
0,396339729	3alpha-HYDROXY-3-DEOXYANGOLENSIC ACID METHYL ESTER
0,395630599	ATORVASTATIN CALCIUM
0,393765457	1-AMINOCYCLOBUTANE CARBOXYLIC ACID
0,392698619	2,6-DI-t-BUTYL-4-METHYLPHENOL
0,392625125	SKATOLE
0,391400597	DIPTERYXIN
0,390457057	SOLANESYL ACETATE
0,388344294	2',5'-DIHYDROXY-4-METHOXYCHALCONE
0,386783286	HESPERIDIN
0,38644202	ALLANTOIN
0,386427276	1-(2-TRIFLUOROMETHYLPHENYL)IMIDAZOLE
0,384989099	MEPHENESIN
0,383360841	TETRANDRINE
0,381169953	3-OXOURSAN (28-13)OLIDE
0,380780719	HEXAMETHYLQUERCETAGETIN
0,380100672	DEHYDROABIETAMIDE
0,379361842	SULFANILAMIDE
0,379045748	INOSINE
0,378946967	GALANTHAMINE HYDROBROMIDE
0,378782695	NIFEDIPINE
0,378679469	HUMULENE (alpha)
0,376661278	PIPERACILLIN SODIUM
0,369012466	RETINOL
0,368303764	PEUCENIN

0,366507037	RANOLAZINE
0,365382783	PHENYL AMINOSALICYLATE
0,363879941	SPAGLUMIC ACID
0,362546946	ETHYL EVERNINATE
0,359038784	GLUCOSAMINIC ACID
0,359029648	DROPERIDOL
0,358596315	RITODRINE HYDROCHLORIDE
0,355850546	2',4'-DIHYDROXYCHALCONE 4'-GLUCOSIDE
0,355823701	ARECOLINE HYDROBROMIDE
0,35401115	TILORONE
0,352422009	TRIACETYLRESVERATROL
0,352102954	AMINOGLUTETHIMIDE
0,351279421	BRUCINE
0,350999437	METHYLXANTHOXYLIN
0,350867618	AMRINONE
0,349761344	3beta-HYDROXY-23,24-BISNORCHOL-5-ENIC ACID
0,349042117	PROADIFEN HYDROCHLORIDE
0,348773065	2-METHOXY-5 (6)EPOXY-TETRAHYDROCARYOPHYLLENE
0,34737953	HARMALINE
0,347180263	DICYCLOMINE HYDROCHLORIDE
0,346453151	AMITRAZ
0,345774207	KETOROLAC TROMETHAMINE
0,343819177	CEPHRADINE
0,343436544	OXACILLIN SODIUM
0,343036624	RUBESCENSIN A
0,341687879	GLIPIZIDE
0,341039637	BROMHEXINE HYDROCHLORIDE
0,338319039	ZOPICLONE
0,33672443	3-BROMO-7-NITROINDAZOLE
0,336010033	COTININE
0,335294298	AMINOHIPPURIC ACID
0,334784498	EVOXINE
0,334540442	CYPROTERONE
0,333163226	UBIDECARENEONE
0,332609332	INDAPAMIDE
0,332374965	PHTHALYLSULFATHIAZOLE
0,330044532	TRIMETHOPRIM
0,329698514	REPAGLINIDE
0,328731645	2-METHYL-4-(PIPERIDIN-1-YLCARBOXY)-5-ISOPROPYLPHENYLTRIMETHYLAMMONIUM CHLORIDE
0,328657603	CINOXACIN
0,327712866	AMIFOSTINE
0,327289445	NORSTICTIC ACID
0,326378024	BUTYL PARABEN
0,325988622	MEPENZOLATE BROMIDE
0,324979209	ACETOSYRINGONE
0,324199799	NEFOPAM
0,323832765	DIENESTROL
0,322766939	RIMANTADINE HYDROCHLORIDE
0,32271576	TRIGONELLINE
0,322073335	ANETHOLE
0,321639611	NETILMICIN SULFATE
0,320920399	CHOLEST-5-EN-3-ONE

0,320587507	PREGNENOLONE SULFATE, SODIUM SALT
0,317485885	PHLORIDZIN
0,316994881	DIOSMIN
0,316888828	IODIPAMIDE
0,315277599	RESORCINOL MONOACETATE
0,313934292	IOPANIC ACID
0,310519897	CYCLOCREATINE
0,309984464	N-METHYLANTHRANILIC ACID
0,308439218	KASUGAMYCIN HYDROCHLORIDE
0,307654322	TOLAZAMIDE
0,303803397	NOBILETIN
0,302653501	EPIAFZELECHIN (2R,3R)(-)
0,301368796	ETHYLNOREPINEPHRINE HYDROCHLORIDE
0,300461252	CELECOXIB
0,300405176	6alpha-METHYLPREDNISOLONE ACETATE
0,300142883	HARMANE
0,297303613	METOCLOPRAMIDE HYDROCHLORIDE
0,297227311	3alpha-ACETOXYDIHYDRODEOXYGEDUNIN
0,296385664	IRBESARTAN
0,295455965	DAIDZEIN
0,293324282	CETYLPYRIDINIUM CHLORIDE
0,293105566	ASIATIC ACID
0,29176073	BEPHENIUM HYDROXYNAPTHOATE
0,291350402	SULFAPHENAZOLE
0,290409576	NALTREXONE HYDROCHLORIDE
0,28981603	BILIRUBIN
0,28962158	BLEOMYCIN (bleomycin B2 shown)
0,287227632	SULFISOXAZOLE ACETYL
0,286547162	LAGOCHILIN
0,286504714	LARIXINIC ACID
0,285595774	FRAXIDIN METHYL ETHER
0,285582635	IRIGENIN
0,285085474	EPIANDROSTERONE
0,28477178	POMIFERIN
0,283170889	CARISOPRODOL
0,282885764	PAPAVERINE HYDROCHLORIDE
0,282679205	TRIAMCINOLONE DIACETATE
0,28145106	HEDERAGENIN
0,281449535	CEFUROXIME AXETIL
0,280643593	PIRENZEPINE HYDROCHLORIDE
0,279704683	ROTENONIC ACID, METHYL ETHER
0,278636693	PHENACETIN
0,27794133	QUINAPRIL HYDROCHLORIDE
0,277933782	METARAMINOL BITARTRATE
0,270580427	TROPICAMIDE
0,26950152	DIRITHROMYCIN
0,265011101	MUPIROCIN
0,264835603	LOGANIN
0,263486474	XANTHYLETIN
0,262779587	BACCATIN III
0,261569644	LIMONIN
0,259517926	THERMOPSINE PERCHLORATE

0,25852951	FLUROTHYL
0,257191084	BENFLURALIN
0,256524097	SPERMINE
0,255484708	FENOPROFEN
0,255384521	SALSOLIDINE
0,254939005	PHENYTOIN SODIUM
0,254894337	SALICIN
0,253475568	FIPRONIL
0,253106183	SR-2640
0,251834709	ESTRADIOL DIACETATE
0,251493507	p-CHLOROPHENYLALANINE
0,24923149	S-ISOCORYDINE (+)
0,248788346	PRIMULETIN
0,247476566	SULPIRIDE
0,247434844	SULFAGUANIDINE
0,245560692	DEMETHYLNOBILETIN
0,244504498	TRIMIPRAMINE MALEATE
0,242154467	BENZOYLPAS
0,241462052	VENLAFAXINE
0,237438627	SULCONAZOLE NITRATE
0,23486484	NADOLOL
0,234519152	HINOKITIOL
0,231201246	2-ACETYLPYRROLE
0,230938672	CHRYSAROBIN
0,230938672	2,4-DICHLOROPHENOXYBUTYRIC ACID
0,229886312	ENILCONAZOLE
0,227614308	NORETHINDRONE
0,226435843	CARBOPLATIN
0,224878585	HYDROXYPROGESTERONE
0,224550079	COSMOSIIN
0,224436993	GATIFLOXACIN
0,223880186	MODAFINIL
0,223653592	ACEPROMAZINE MALEATE
0,220987618	POTASSIUM p-AMINOBENZOATE
0,220471308	LANOSTEROL ACETATE
0,21937978	CLONIDINE HYDROCHLORIDE
0,218949867	LINCOMYCIN HYDROCHLORIDE
0,218054846	DROFENINE HYDROCHLORIDE
0,217203292	DICHLORVOS
0,2169798	METHYLENE BLUE
0,216122138	NORSTICTIC ACID TRIACETATE
0,21401789	CHLORPROPAMIDE
0,213137665	AZAPERONE
0,212075286	LOBENDAZOLE
0,210287605	2,5-DI-t-BUTYL-4-HYDROXYANISOLE
0,209588959	N-PHENYLANTHRANILIC ACID
0,207583315	GABOXADOL HYDROCHLORIDE
0,206781797	PUERARIN
0,203491629	N- (9-FLUORENYLMETHOXYCARBONYL)-L-LEUCINE
0,200747479	PENTOBARBITAL
0,200735124	MEXILETINE HYDROCHLORIDE
0,199191566	1-HYDROXY-3,6,7-TRIMETHOXY-2,8-DIPRENYLXANTHONE

0,199055689	FENOFIBRATE
0,198927255	STROPHANTHIDINIC ACID LACTONE ACETATE
0,197890614	PROGLUMIDE
0,196941783	PHENYLETHYL ALCOHOL
0,19692952	NAFRONYL OXALATE
0,19486217	IFOSFAMIDE
0,193797208	MENADIONE
0,192556328	CHLORMADINONE ACETATE
0,189274864	THIOTHIXENE
0,184793717	PANTOTHENIC ACID(d) Na salt
0,183575861	BROMOCRIPTINE MESYLATE
0,181559514	FRAXETIN
0,178366774	BROMINDIONE
0,176330318	ROLITETRACYCLINE
0,174709687	ORLISTAT
0,174591699	METHAMIDOPHOS
0,173110612	RIFAXIMIN
0,173029179	OMEGA-3-ACID ESTERS (EPA shown)
0,171305414	PTAEROXYLIN
0,168023505	SEROTONIN HYDROCHLORIDE
0,166415454	AMINOCAPROIC ACID
0,166281056	AURIN TRICARBOXYLIC ACID
0,165956319	XYLOSE
0,164815526	ERYSOLIN
0,162930484	TRIMEPRAZINE TARTRATE
0,16204873	AESCULIN
0,161836477	GALLIC ACID
0,161727315	KARANJIN
0,161222452	2,2'-AZO-bis-2-AMINOPROPANE
0,160626905	7-OXOCHOLESTEROL
0,157916365	RIBOSTAMYCIN SULFATE
0,157774318	PHLORETIN
0,157203828	ISOTRETINON
0,157063815	PHENYLBUTYRIC ACID
0,156685597	PHLORACETOPHENONE
0,155924976	2',3-DIHYDROXY-4,4',6'-TRIMETHOXYCHALCONE
0,153564902	HOMOPTEROCARPIN
0,153547163	TETRAHYDROSAPPANONE A TRIMETHYL ETHER
0,152787042	URETHANE
0,15033542	ACTINONIN
0,150260488	OXONITINE
0,149502366	3-BROMO-3,4,4-TRIMETHYL-3,4-DIHYDRODIAZETE-1.2-DIOXIDE
0,1493924	OFLOXACIN
0,148390214	SUCRALOSE
0,147390983	MINOXIDIL
0,144913835	CEFUROXIME SODIUM
0,14344553	TUAMINOHEPTANE SULFATE
0,142579671	CHLORPROMAZINE
0,140924165	DEOXYSAPPANONE B TRIMETHYL ETHER
0,135921346	VALSARTAN
0,131953801	2-METHYL GRAMINE
0,131716041	FOSFOSAL

0,13126826	ATOVAQUONE
0,1302895	ZIDOVUDINE [AZT]
0,128741798	FLUMEQUINE
0,128498043	FLUCYTOSINE
0,126877053	KYNURENINE
0,126628613	TERFENADINE
0,126202432	INDOPROFEN
0,125654682	MECAMYLAMINE HYDROCHLORIDE
0,125266689	RIBAVIRIN
0,1246955	BOVINOCIDIN (3-nitropropionic acid)
0,123638315	2-METHYLENE-5-(2,5-DIOXOTETRAHYDROFURAN-3-YL)-6-OXO--10,10-DIMETHYLBICYCLO[7: 2: 0]UNDECANE
0,123272986	MITOMYCIN C
0,122136175	3,5-DIHYDROXYFLAVONE
0,120808673	VIOLASTYRENE
0,119695019	METHAPYRILENE HYDROCHLORIDE
0,11932138	DALBERGIONE
0,116867887	TILMICOSIN
0,115797728	LEVOCARNITINE
0,115395955	BAMBUTEROL HYDROCHLORIDE
0,115242081	METHYLATROPINE NITRATE
0,11491967	CONESSINE
0,11253877	LOSARTAN
0,112070758	DINITOLMIDE
0,111460324	SULFADIMETHOXINE
0,111178284	PALMATINE
0,110945654	SULFANILATE ZINC
0,109922947	ERYTHROMYCIN ESTOLATE
0,10858744	2',4-DIHYDROXYCHALCONE
0,108423721	2',4'-DIHYDROXY-4-METHOXYCHALCONE
0,107602316	METAMPICILLIN SODIUM
0,107284933	BAICALEIN
0,106813978	7-HYDROXYETHYLTHEOPHYLLINE
0,106680382	CARBAMAZEPINE
0,10651748	PRACTOLOL
0,10550281	MILTEFOSINE
0,104655604	VECURONIUM BROMIDE
0,104576267	PILOCARPINE NITRATE
0,104570686	LEVOBUNOLOL HYDROCHLORIDE
0,100417798	D-LACTITOL MONOHYDRATE
0,098965775	CEFOPERAZONE SODIUM
0,09804691	BETULIN
0,097882589	BISANHYDRORUTILANTINONE
0,096885225	BENDROFLUMETHIAZIDE
0,096736289	CLOPIDOL
0,096192957	PIROMIDIC ACID
0,096015049	ACETRIAZOIC ACID
0,094468227	PAEONOL
0,093642077	TRIMETHOBENZAMIDE HYDROCHLORIDE
0,093585295	TENIPOSIDE
0,09333534	THIORIDAZINE HYDROCHLORIDE
0,090884743	HAEMATOMMIC ACID
0,090246198	CETIRIZINE HYDROCHLORIDE

0,089687399	ANTIAROL
0,089298199	NICERGOLINE
0,087687334	TOLPERISONE HYDROCHLORIDE
0,087270708	MORANTEL CITRATE
0,084070186	CARBINOXAMINE MALEATE
0,081443281	GRISEOFULVIC ACID
0,081401825	ESTRONE ACETATE
0,080391379	URSINOIC ACID
0,080113146	IRIGENIN TRIMETHYL ETHER
0,078103938	2,3-DIMERCAPTOSUCCINIC ACID
0,077215779	DICUMAROL
0,076726389	TOBRAMYCIN
0,076699224	FLUOXETINE
0,073016893	PERGOLIDE MESYLATE
0,070863261	ENOXAPARIN SODIUM (1% wt/vol in 10%aq DMSO)
0,07071235	BROMPHENIRAMINE MALEATE
0,070680425	ETOMIDATE
0,07051392	DICHLOBENIL
0,07047742	CLIDINIUM BROMIDE
0,069815795	DEHYDROCHOLIC ACID
0,069780155	STIGMASTEROL
0,069521055	ENROFLOXACIN
0,069440239	BUCLADESINE
0,068668586	KETOCONAZOLE
0,067681585	MONOCROTALINE
0,064279045	ECONAZOLE NITRATE
0,064178685	COLESEVALAM HYDROCHLORIDE (high mol wt copolymer @10mg/ml)
0,062927989	AVOBENZONE
0,062108904	CEFTIBUTEN
0,059878508	PREGNENOLONE
0,059517787	XANTHURENIC ACID
0,058811268	SULOCTIDIL
0,058309841	CLAVULANATE LITHIUM
0,056063899	CHOLESTAN-3beta,5alpha,6beta-TRIOL
0,055884439	CEFAMANDOLE NAFATE
0,055497479	ARISTOLOCHIC ACID
0,052969929	EPICATECHIN PENTAACETATE
0,051343428	2,3,4-TRIHYDROXY-4'-ETHOXYBENZOPHENONE
0,050794731	BENZOIC ACID
0,050177412	BORNEOL
0,050155593	SIROLIMUS
0,049265212	THIAMINE
0,048200279	GOSSYPIN
0,047971108	TRANYLCYPROMINE SULFATE
0,04756213	NARINGIN
0,047445929	PRILOCAINE HYDROCHLORIDE
0,046040015	ACACETIN
0,044816964	MENTHONE
0,04343141	FOMEPIZOLE HYDROCHLORIDE
0,043110211	CITICOLINE
0,043079177	TRANEXAMIC ACID
0,043039266	FLUFENAMIC ACID

0,040862986	MORIN
0,040471742	SALVINORIN A
0,04042943	ZINC UNDECYLENATE
0,040419559	ANABASINE HYDROCHLORIDE
0,040196948	HARPAGOSIDE
0,039902755	METAMECONINE
0,039591946	QUINOLINIC ACID
0,038991309	OLANZAPINE
0,038990081	AMINOCYCLOPROPANECARBOXYLIC ACID
0,038083456	THIOSTREPTON
0,037826176	DONEPEZIL HYDROCHLORIDE
0,035631431	MEBEVERINE HYDROCHLORIDE
0,035336258	BETAMIPRON
0,033362022	TRIHEXYPHENIDYL HYDROCHLORIDE
0,031318592	PERINDOPRIL ERBUMINE
0,02997906	18alpha-GLYCYRRHETINIC ACID
0,029296406	S-(1,2-DICARBOXYETHYL)GLUTATHIONE
0,028030354	CEFDINIR
0,02532542	OXAPROZIN
0,025207037	NIACIN
0,024299924	CINCHONINE
0,024081651	SULFACHLORPYRIDAZINE
0,021759897	FENDILINE HYDROCHLORIDE
0,021179015	LOBELINE HYDROCHLORIDE
0,019991979	CHLORMEZANONE
0,01497422	TAURINE
0,012420299	DIHYDROCELASTROL
0,010671573	AMINOBENZTROPINE
0,010551421	HARMOL HYDROCHLORIDE
0,010210763	QUERCETIN PENTAMETHYL ETHER
0,00486927	ISOGUVACINE HYDROCHLORIDE
0,004373181	GEDUNOL
0,002105881	CAPSAICIN
0,001810093	2',4'-DIHYDROXYCHALCONE
0,001736166	FAMPRIDINE
0,00141029	RONNEL
0,000255682	TUBOCURARINE CHLORIDE
0,000150577	PINDOLOL
-7,94789E-06	TOLBUTAMIDE
-0,000972815	PREDNISOLONE SODIUM PHOSPHATE
-0,001513458	CEFOTAXIME SODIUM
-0,002387538	DEACETOXY(7)-7-OXOKHIVORINIC ACID
-0,003054162	AZLOCILLIN SODIUM
-0,004198694	TRICHLORMETHIAZIDE
-0,006296287	BENURESTAT
-0,006856926	6,4'-DIMETHOXYFLAVONE
-0,007522289	PTERYXIN
-0,008787464	MYOSMINE
-0,010044743	BRAZILEIN
-0,010128784	PARTHENOLIDE
-0,010483774	DIGOXIGENIN
-0,012806579	SURAMIN

-0,013176027	METHYSERGIDE MALEATE
-0,014317764	DERACOXIB
-0,014368318	ENALAPRIL MALEATE
-0,014421823	NORCANTHARIDIN
-0,014901113	PRAMOXINE HYDROCHLORIDE
-0,016337644	TRIFLUOPERAZINE HYDROCHLORIDE
-0,016863833	DIHYDROGEDUNINIC ACID, METHYL ESTER
-0,016883191	FOLIC ACID
-0,019852019	7,4'-DIMETHOXYISOFLAVONE
-0,020782641	RANITIDINE
-0,021415447	PROPAZINE
-0,023647221	DESACETYLCOLFORSIN
-0,023944868	RIBOFLAVIN
-0,025732615	TIOCONAZOLE
-0,026984375	DICHLOROPHENE
-0,027618382	ROSMARINIC ACID
-0,027681973	CHOL-11-ENIC ACID
-0,030602545	TICARCILLIN DISODIUM
-0,030972308	MONENSIN SODIUM (monensin A is shown)
-0,031034433	TRIFLURIDINE
-0,031915838	PYRIDOXINE
-0,033557567	ISOSORBIDE MONONITRATE
-0,034129042	ACEDAPSONE
-0,035162339	PHYTOL
-0,037779224	CEFAMANDOLE SODIUM
-0,040188756	RONIDAZOLE
-0,040920378	ISOFORMONONETIN
-0,041629849	FLUDARABINE PHOSPHATE
-0,042382165	ARSANILIC ACID
-0,042496798	GLICLAZIDE
-0,043335687	SHIKIMIC ACID
-0,044256773	ADRENALINE BITARTRATE
-0,045026105	BISMUTH SUBSALICYLATE
-0,046433861	METHYSERGIDE
-0,047491492	OLMESARTAN MEDOXOMIL
-0,050060725	BUMETANIDE
-0,050791753	MEGLUMINE
-0,052011665	3-DEACETYLKHIVORIN
-0,052932778	CHUKRASIN METHYL ETHER
-0,055739148	HELICIN
-0,056496177	PICROPODOPHYLLOTOXIN
-0,057627299	CHLORZOXAZONE
-0,058295876	DEHYDROROTENONE
-0,060655792	CAFFEINE
-0,060716202	FISSINOLIDE
-0,062318814	ARTEMISININ
-0,062600296	CAMPHOR (1R)
-0,062962755	4-METHYLDAPHNETIN
-0,06315335	CREATININE
-0,06326788	BUCETIN
-0,063351258	L-ALANINOL
-0,066396415	TANSHINONE IIA SULFONATE SODIUM

-0,067335754	BETAINE HYDROCHLORIDE
-0,067804856	PANTHENOL
-0,068293823	QUIPAZINE MALEATE
-0,069434341	PIMOZIDE
-0,069903905	THIOTEPA
-0,071612959	DIMENHYDRINATE
-0,072878044	PHENYLBUTYRATE SODIUM
-0,073668385	ASCORBIC ACID
-0,073889672	THIAMPHENICOL
-0,073968192	EPICATECHIN MONOGALLATE
-0,074017861	UNDECYLENIC ACID
-0,07481207	THALIDOMIDE
-0,074931783	2',4'-DIHYDROXY-6'-METHOXYACETOPHENONE
-0,07547391	ERGOSTEROL
-0,07789739	NICOTINYL ALCOHOL TARTRATE
-0,07805755	HYDROXYTACRINE MALEATE
-0,07811903	NIMUSTINE
-0,079481191	alpha-CYANO-3-HYDROXYCINNAMIC ACID
-0,079996751	MUNDOSERONE
-0,080265495	SUCCINYLSULFATHIAZOLE
-0,080634505	CEFPODOXIME PROXETIL
-0,081843657	PIOGLITAZONE HYDROCHLORIDE
-0,082169119	CISPLATIN
-0,083566118	BUPROPION
-0,08465319	PIRENPERONE
-0,086082427	LITHIUM CITRATE
-0,086166822	NORSTICTIC ACID PENTAACETATE
-0,088555406	21-ACETOXYPREGNENOLONE
-0,08877376	5alpha-ANDROSTAN-3,17-DIONE
-0,089082291	ATENOLOL
-0,089180884	PYRAZINAMIDE
-0,090120242	7,2'-DIHYDROXYFLAVONE
-0,090126144	7,8-DIHYDROXYFLAVONE
-0,091737684	DIFUCOL HEXAMETHYL ETHER
-0,091985321	DIACERIN
-0,092111656	4,4'-DIMETHOXYDALBERGIONE
-0,094062065	SANTONIN
-0,09431004	DICLOXACILLIN SODIUM
-0,094547022	LEVAMISOLE HYDROCHLORIDE
-0,095837574	SULFAMETER
-0,097835286	LORATADINE
-0,09885988	CYCLOTHIAZIDE
-0,099047807	SUPROFEN METHYL ESTER
-0,099200389	ETHOSUXIMIDE
-0,099892703	ROCCELLIC ACID
-0,100871953	CEFSULODIN SODIUM
-0,101487498	CEPHALOSPORIN C SODIUM
-0,104080302	beta-SITOSTEROL
-0,10435332	ARBUTIN
-0,105521947	PANCURONIUM BROMIDE
-0,107133952	TICLOPIDINE HYDROCHLORIDE
-0,107219027	ETHAMIVAN

-0,110085633	HEXAMETHONIUM BROMIDE
-0,110505292	ANGOLENSIN (R)
-0,110959832	THEAFLAVIN
-0,111561092	XANTHOPTERIN
-0,112341542	BENZYL ISOTHIOCYANATE
-0,114369742	OXELAIDIN CITRATE
-0,115320258	STROPHANTHIDIN
-0,119242892	CAPREOMYCIN SULFATE
-0,120024053	DANTROLENE SODIUM
-0,120346833	BUSULFAN
-0,120745252	DIPERODON HYDROCHLORIDE
-0,120871831	TRIMETOZINE
-0,122592361	DEOXYADENOSINE
-0,124151812	ETHOXZOLAMIDE
-0,124346798	FOSCARNET SODIUM
-0,125737946	METHYLPHENIDATE HYDROCHLORIDE
-0,130090883	ORSELLINIC ACID, ETHYL ESTER
-0,130100793	FENSPIRIDE HYDROCHLORIDE
-0,130719841	MEFLOQUINE
-0,131152417	6-PHENYL-4-AZOBICYCLO[5.4.0]UNDECA-7,9,11-TRIENE-9,10-DIOL
-0,131830012	PERPHENAZINE
-0,13184153	MEPIVACAINE HYDROCHLORIDE
-0,133425075	10-HYDROXYCAMPTOTHECIN
-0,134354182	BUTACAINE
-0,136596731	PANTETHINE
-0,13670294	CARNOSIC ACID
-0,136814258	AVOCADANOFURAN
-0,138385875	2,6-DIHYDROXY-4-METHOXYTOLUENE
-0,138557656	EUPARIN
-0,138848632	NAPROXOL
-0,139656269	ERGOSTEROL ACETATE
-0,139814666	TIAPRIDE HYDROCHLORIDE
-0,141841413	DICHLORISONE ACETATE
-0,141902207	CRUSTECDYSONE
-0,144900841	RALOXIFENE HYDROCHLORIDE
-0,147297047	METERGOLINE
-0,147875772	KANAMYCIN A SULFATE
-0,148104697	LUPEOL
-0,150764816	DOXAZOSIN MESYLATE
-0,152394431	GAMBOGIC ACID AMIDE
-0,152833703	SIBUTRAMINE HYDROCHLORIDE
-0,153392431	STRYCHNINE METHIODIDE
-0,156137243	ETOPOSIDE
-0,156371403	SECNIDAZOLE
-0,156454888	TACROLIMUS
-0,156503713	ALEXIDINE HYDROCHLORIDE
-0,159984626	TRICLOSAN
-0,160333207	DIHYDROGAMBOGIC ACID
-0,16059649	PIMPINELLIN
-0,161460566	NIACINAMIDE
-0,162324276	ESTRADIOL BENZOATE
-0,163676311	DIOXYBENZONE

-0,167073611	CARPROFEN
-0,167303826	OLEANOLIC ACID ACETATE
-0,167477715	PHENFORMIN HYDROCHLORIDE
-0,168426269	MESNA
-0,168568336	MEPHENTERMINE SULFATE
-0,168578102	CEFTAZIDIME
-0,16950759	LAMOTRIGINE
-0,171492838	DICHLOROPROP
-0,171801548	HOMOSALATE
-0,173212924	SACCHARIN
-0,175574121	2-BENZOYL-5-METHOXYBENZOQUINONE
-0,176349609	FIPEXIDE HYDROCHLORIDE
-0,176552523	PACHYRRHIZIN
-0,1767507	4-METHOXYDALBERGIONE
-0,176913318	NEROL
-0,178276386	ACEMETACIN
-0,180347687	PANGAMIC ACID SODIUM
-0,182197942	LAWSONE
-0,182995229	PERILLIC ACID (-)
-0,185656721	ZOLMITRIPTAN
-0,18586041	7beta-HYDROXY-7-DESACETOXYKHIVORINIC ACID, METHYL ESTER
-0,186124603	CEPHALEXIN
-0,18615097	CITALOPRAM
-0,187586726	FELBAMATE
-0,187897369	MIDODRINE HYDROCHLORIDE
-0,189385912	PANTOPRAZOLE
-0,190778925	PROPRANOLOL HYDROCHLORIDE (+/-)
-0,191760772	CARYLOPHYLLENE OXIDE
-0,194051071	2,6-DIMETHOXYQUINONE
-0,194629933	OXOLINIC ACID
-0,19584831	CYCLOBENZAPRINE HYDROCHLORIDE
-0,195998175	ISOXICAM
-0,197581363	PECTOLINARIN
-0,19767903	ENOXACIN
-0,197984093	BERGAPTOL
-0,19911282	CLORSULON
-0,199417872	GLUCONOLACTONE
-0,20227059	DIHYDROJASMONIC ACID
-0,202487769	AMINOPENTAMIDE
-0,202864633	NARINGENIN
-0,203435917	DAPHNETIN
-0,204360977	ROXITHROMYCIN
-0,204530331	LABETALOL HYDROCHLORIDE
-0,205263803	CINNARAZINE
-0,205362845	PERHEXILINE MALEATE
-0,206576322	EUPHOL
-0,206757641	METHACHOLINE CHLORIDE
-0,207065353	ALAPROCLATE
-0,207309674	ESTROPIPATE
-0,207672836	GRAYANOTOXIN I
-0,211733023	DIATRIZOIC ACID
-0,213139037	ANISODAMINE

-0,214121259	METHAZOLAMIDE
-0,214352744	SMILAGENIN
-0,214911836	GEMIFLOXACIN MESYLATE
-0,217104775	CLOPIDOGREL SULFATE
-0,217755178	L-DEOXYALLIIN
-0,220487052	PERUVOSIDE
-0,220747235	CEFMETAZOLE SODIUM
-0,222773389	ALENDRONATE SODIUM
-0,223219769	NATEGLINIDE
-0,224189327	DESMETHYLDIHYDROCAPSAICIN
-0,224500472	6,7-DIHYDROXYFLAVONE
-0,224975224	6,2'-DIMETHOXYFLAVONE
-0,225432813	ETODOLAC
-0,225864683	DIHYDROXY (3alpha,12alpha)PREGNAN-20-ONE
-0,2272475	ALPINETIN METHYL ETHER
-0,22750384	ISOTECTORIGENIN, 7-METHYL ETHER
-0,229984342	ALFLUZOSIN
-0,23070402	AMINOLEVULINIC ACID HYDROCHLORIDE
-0,232818882	BENZOYL PEROXIDE
-0,234153315	CIPROFLOXACIN
-0,234753907	TRIDESACETOXYKHIVORIN
-0,236640499	GARCINOLIC ACID
-0,236815821	CAPOBENIC ACID
-0,236888217	SAFROLE
-0,237745219	BERBERINE CHLORIDE
-0,238506213	SITAGLIPTIN
-0,238536395	FLOPROPIONE
-0,239739086	DECOQUINATE
-0,241931975	TOLFENAMIC ACID
-0,241987595	NISOLDIPINE
-0,242771205	NONIC ACID
-0,24327779	METHOPRENE (S)
-0,245191343	ASTRAGALOSIDE IV
-0,245946023	THONZYLAMINE HYDROCHLORIDE
-0,246022982	DALBERGIONE, 4-METHOXY-4'-HYDROXY-
-0,246547028	NIZATIDINE
-0,246691126	EDOXUDINE
-0,247950197	PAROXETINE HYDROCHLORIDE
-0,249079991	ACETYL TYROSINE ETHYL ESTER
-0,249897974	CHLOROXINE
-0,251390535	NOSCAPINE HYDROCHLORIDE
-0,252544876	PIRACETAM
-0,252660791	4'-HYDROXYCHALCONE
-0,253872396	ADIPHENINE HYDROCHLORIDE
-0,254788583	TETRAMIZOLE HYDROCHLORIDE
-0,257106991	NILUTAMIDE
-0,25846572	IMPERATORIN
-0,258841168	2,4,5-TRICHLOROPHENOXYACETIC ACID, METHYL ESTER
-0,259581104	EPI(13)TORULOSOL
-0,25978539	ISOSAFROLE
-0,262720589	KOPARIN
-0,263426429	beta-CARYOPHYLLENE ALCOHOL

-0,264978511	BENZYL BENZOATE
-0,264997725	PHTHALYSULFATHIAZOLE
-0,265080312	CORYNANTHINE
-0,266404411	3,4'-DIMETHOXYFLAVONE
-0,268161786	TROLEANDOMYCIN
-0,269677423	2-(N,N-DIETHYLAMINO)DIAZENOLATE-2-OXIDE DIETHYLAMONIUM SALT
-0,269732205	OXIDOPAMINE HYDROCHLORIDE
-0,27004517	11a-ACETOXYPROGESTERONE
-0,27061969	ELLAGIC ACID
-0,270742248	BIPERIDEN
-0,271067795	TERBINAFINE HYDROCHLORIDE
-0,271704447	LEOIDIN
-0,271852686	3-HYDROXYTYRAMINE
-0,272088893	BROXYQUINOLINE
-0,274973801	SENNOSIDE A
-0,27612183	TRIFLUPROMAZINE HYDROCHLORIDE
-0,27676574	DUARTIN, DIMETHYL ETHER
-0,278175502	ACETYLGLUTAMIC ACID
-0,279726571	CHRYSANTHEMIC ACID
-0,280550412	CACODYLIC ACID
-0,283240847	CROTAMITON
-0,283432319	BENZOXYQUINE
-0,284284861	ANCITABINE HYDROCHLORIDE
-0,286244876	ISOPEONOL
-0,286792599	CEFPROZIL
-0,286946754	ALTRENOGEST
-0,287752994	LOPERAMIDE HYDROCHLORIDE
-0,28787389	IRIGENIN, 7-BENZYL ETHER
-0,289775466	NYLIDRIN HYDROCHLORIDE
-0,290701439	CATECHIN PENTAACETATE
-0,290732963	PIPERAZINE
-0,29159765	ELETRIPTAN HYDROBROMIDE
-0,293275131	ETHISTERONE
-0,293899648	PIPERIDOLATE HYDROCHLORIDE
-0,295405713	METHYL PARATHIONE
-0,298046172	VINCAMINE
-0,299162849	BIOTIN
-0,299896146	CARVEDILOL
-0,303099638	CHLOROPHYLLIDE Cu COMPLEX Na SALT
-0,30483786	CYMARIN
-0,305819215	OXFENDAZOLE
-0,306980059	OXYPHENONIUM BROMIDE
-0,308645654	JUGLONE
-0,308848122	FLUORESCEIN
-0,308984957	PYRIDOSTIGMINE BROMIDE
-0,31040729	SILDENAFIL
-0,311731239	CEFDITORIN PIVOXIL
-0,311971439	ALGESTERONE ACETOPHENIDE
-0,313083877	PHENETHYL CAFFEATE (CAPE)
-0,313167878	6,4'-DIHYDROXYFLAVONE
-0,313690766	BEZAFIBRATE
-0,315344596	ACETYLTRYPTOPHANAMIDE

-0,317087216	OSAJIN
-0,317487273	TELMISARTAN
-0,323151157	FLUOROMETHOLONE
-0,323566861	BENFLUOREX HYDROCHLORIDE
-0,323669779	VULPINIC ACID
-0,323866247	DOXYCYCLINE HYDROCHLORIDE
-0,325412289	SERTRALINE HYDROCHLORIDE
-0,325759249	LIOTHYRONINE (L- isomer) SODIUM
-0,326233837	SPIPERONE
-0,326583136	SUMATRIPTAN
-0,327445275	N-METHYLBENZYLAMINE HYDROCHLORIDE
-0,328745646	TINIDAZOLE
-0,328867897	DIETHYLSTILBESTROL
-0,334827415	ABSCISIC ACID (cis,trans; +/-)
-0,335273989	PEONOL METHYL ETHER
-0,335653622	6,7-DICHLORO-3-HYDROXY-2-QUINOXALINECARBOXYLIC ACID
-0,335991791	CITROPTEN
-0,336213952	PEFLOXACINE MESYLATE
-0,336708767	3beta-HYDROXYDEOXODIHYDRODEOXYGEDUNIN
-0,336964273	ACETYLPHENYLALANINE
-0,337267102	ESCULETIN
-0,338521836	MARMESIN ACETATE
-0,339789181	SELAMECTIN
-0,340220309	ALLOXAN
-0,340816599	OXYBENZONE
-0,341283379	ALTRETAMINE
-0,344825874	ITRACONAZOLE
-0,34624556	GANGALEOIDIN
-0,346722281	ASCORBYL PALMITATE
-0,348616595	CYTISINE
-0,348626531	TOPIRAMATE
-0,349617657	N-ACETYLNEURAMIC ACID
-0,351875634	FLOXURIDINE
-0,353990003	HEMATEIN
-0,354103462	AKLAVINE HYDROCHLORIDE
-0,3567316	RABEPRAZOLE SODIUM
-0,361312816	PEONIFLORIN
-0,362108167	KYNURENIC ACID
-0,362157604	DICHLORODIPHENYLTRICHLOROETHANE
-0,362806455	SUCCINYLACETONE
-0,363641546	BENZYDAMINE HYDROCHLORIDE
-0,366258821	ALISKIREN HEMIFUMARATE
-0,367046622	CLIOQUINOL
-0,370400068	EPIAFZELECHIN TRIMETHYL ETHER
-0,371497964	OXANTEL PAMOATE
-0,373141971	DECAHYDROGAMBOGIC ACID
-0,375631628	OXETHAZAINE
-0,376890161	DIHYDROMYRISTICIN
-0,381851362	BETA-PROPIOLACTONE
-0,381984508	PIPAMPERONE
-0,382054877	XANTHOXYLIN
-0,385631957	CLOFAZIMINE

-0,385761012	COLISTIN SULFATE
-0,385821992	TOLTRAZURIL
-0,386304241	3-AMINO-beta-PINENE
-0,387346864	PHENYLMERCURIC ACETATE
-0,387913244	CEFONICID SODIUM
-0,390814648	CYCLOHEXIMIDE
-0,393394814	CAMPTOTHECIN
-0,393805324	ANHYDROBRAZILIC ACID
-0,397210509	GLYCOPYRROLATE
-0,402226126	AGELASINE (stereochemistry of diterpene unknown)
-0,405185043	LACCAIC ACID A
-0,406340185	LOMEFLOXACIN HYDROCHLORIDE
-0,40678194	LEVALBUTEROL HYDROCHLORIDE
-0,407328555	O-BENZYL-l-SERINE
-0,411750581	RAMELTEON
-0,416013913	3,6-DIMETHOXYFLAVONE
-0,416335672	OMEPRAZOLE
-0,417788171	TEICOPLANIN [A(2-1) shown]
-0,420557535	QUERCETIN TETRAMETHYL (5,7,3',4') ETHER
-0,421566801	RIBOFLAVIN 5-PHOSPHATE SODIUM
-0,422339116	BROMO-3-HYDROXY-4-(SUCCIN-2-YL)-CARYOLANE gamma-LACTONE
-0,423850561	CLONAZEPAM
-0,424061229	NATAMYCIN
-0,424072061	APIGENIN DIMETHYL ETHER
-0,425878423	LAPPACONITINE
-0,428857055	FEXOFENADINE HYDROCHLORIDE
-0,42931953	RIZATRIPTAN BENZOATE
-0,4293831	CARMINIC ACID
-0,430398895	EZETIMIBE
-0,432929317	ALANYL-dl-PHENYLALANINE
-0,436080996	6,3'-DIMETHOXYFLAVONE
-0,436759392	GLUCITOL-4-GUCOPYANOSIDE
-0,438428162	HYCANTHONE
-0,439504565	OBTUSAQUINONE
-0,440465712	3-METHYLORSELLINIC ACID
-0,442195627	VERAPAMIL HYDROCHLORIDE
-0,442345978	CHLOROCRESOL
-0,443274961	EUCATROPINE HYDROCHLORIDE
-0,443632103	THYMOQUINONE
-0,444491816	AJMALINE
-0,445267038	QUETIAPINE
-0,445397899	PROPARACAINE HYDROCHLORIDE
-0,44612318	CHLORPROTHIXENE HYDROCHLORIDE
-0,446458814	GIBBERELLIC ACID
-0,446578953	CELLOBIOSE (D[+])
-0,450488268	MEBHYDROLIN NAPHTHALENESULFONATE
-0,451584355	DIMAPRIT DIHYDROCHLORIDE
-0,452824872	FINASTERIDE
-0,454687373	PIZOTYLINE MALATE
-0,461413094	FORMESTANE
-0,463076085	ACACETIN DIACETATE
-0,464662723	GLYPHOSATE

-0,466491344	OXICONAZOLE NITRATE
-0,467528469	METFORMIN HYDROCHLORIDE
-0,467729126	13-METHYL-4,4-BISNOR-8,11,13-PODOCARPATRIEN-3-ONE
-0,470984059	QUERCITRIN
-0,473568836	THIOCTIC ACID
-0,475969311	IRETOL
-0,479769534	LOBARIC ACID
-0,480090994	NAPROXEN(+)
-0,481628471	LECANORIC ACID
-0,482491834	FISETIN
-0,482602374	TETRAC
-0,483715944	DILOXANIDE FUROATE
-0,484015645	ORNIDAZOLE
-0,484653486	HALOTHANE
-0,485163529	CEFTRIAXONE SODIUM TRIHYDRATE
-0,486474337	PENFLURIDOL
-0,486592028	DIHYDROTANSHINONE I
-0,487290566	MESALAMINE
-0,496217168	CHLORTHALIDONE
-0,496616906	TETRAHYDROPALMATINE
-0,498094452	PARAMETHADIONE
-0,499694526	KOBUSONE
-0,500923452	SCOPOLETIN
-0,502565298	AMYGDALIN
-0,508148038	CELASTROL
-0,510472791	ETIDRONATE DISODIUM
-0,517921741	ESTRADIOL-3-SULFATE, SODIUM SALT
-0,518760851	PHENOTHRIN
-0,521909676	CHLORAMPHENICOL HEMISUCCINATE
-0,524000624	NEROLIDOL
-0,525109229	NITRENDIPINE
-0,529369028	ABIETIC ACID
-0,529770569	ABRINE (L)
-0,530452164	ACAMPROSATE CALCIUM
-0,53414015	CADAVERINE TARTRATE
-0,53792715	PREGNENOLONE SUCCINATE
-0,539957521	18-AMINOABIETA-8,11,13-TRIENE SULFATE
-0,543758464	FLORFENICOL
-0,543860107	AVOCADENOFURAN
-0,544164895	L-LEUCYL-L-ALANINE
-0,54448484	DUARTIN (-)
-0,548924583	BERGAPTEN
-0,549642206	TULOBUTEROL
-0,5513204	THEAFLAVIN DIGALLATE
-0,551560126	ABAMECTIN
-0,553086505	CAMYLOFINE DIHYDROCHLORIDE
-0,55310403	4-METHYLESCULETIN
-0,555586342	OXYQUINOLINE HEMISULFATE
-0,556067657	NONOXYNOL-9
-0,557330618	AZELASTINE HYDROCHLORIDE
-0,560462267	CHRYSANTHEMYL ALCOHOL
-0,567031092	2-HYDROXY-3,4-DIMETHOXYBENZOIC ACID

-0,567585258	CARNITINE (dl) HYDROCHLORIDE
-0,567722534	RESVERATROL
-0,567960858	gamma-AMINOBUTYRIC ACID
-0,568262444	N-HYDROXYMETHYLNICOTINAMIDE
-0,570180261	FUMAZENIL
-0,570618298	ISOBUTYLMETHYLXANTHINE
-0,572024106	EUPHORBIASTEROID
-0,574327693	ANABASAMINE HYDROCHLORIDE
-0,57505189	AMIPRILOSE
-0,575412583	TRIENTINE HYDROCHLORIDE
-0,578672326	ARCAINE SULFATE
-0,579846249	STIGMASTA-4,22-DIEN-3-ONE
-0,580032565	NIPECOTIC ACID
-0,581673376	APRAMYCIN
-0,583882336	CANDESARTAN CILEXTIL
-0,585456033	DIGITONIN
-0,587201083	ETHYNODIOL DIACETATE
-0,590971027	DIETHYLTOLUAMIDE
-0,593915863	MANDELIC ACID, METHYL ESTER
-0,5959848	RHETSININE
-0,600149933	VARDENAFIL HYDROCHLORIDE
-0,602553283	ZIMELDINE HYDROCHLORIDE
-0,603486277	ISOETHARINE MESYLATE
-0,60555267	TETRAHYDROGAMBOGIC ACID
-0,605734443	7-DESHYDROXYPYROGALLIN-4-CARBOXYLIC ACID
-0,608077971	CADIN-4-EN-10-OL
-0,610066867	HAEMATOXYLIN
-0,611518749	12a-HYDROXY-9-DEMETHYLMUNDUSERONE-8-CARBOXYLIC ACID
-0,612879233	ACETYLGLUCOSAMINE
-0,613036104	TENOXICAM
-0,616853418	PHENYLEPHRINE HYDROCHLORIDE
-0,617101076	SALINOMYCIN, SODIUM
-0,617130357	CANTHARIDIN
-0,62282186	DESACETYL (7)KHIVORINIC ACID, METHYL ESTER
-0,630180065	ETHAVERINE HYDROCHLORIDE
-0,633640176	3-METHYLXANTHINE
-0,635158309	TACRINE HYDROCHLORIDE
-0,637322558	DIMETHADIONE
-0,64506995	METOLAZONE
-0,646757465	VERATRIC ACID
-0,647275124	DENATONIUM BENZOATE
-0,649078219	AMIODARONE HYDROCHLORIDE
-0,649450225	ROTENONE
-0,649842553	APHYLLIC ACID
-0,651573169	BENZBROMARONE
-0,652671447	KYNURAMINE
-0,654049242	TOMATINE
-0,654237663	LEVULINIC ACID, 3-BENZYLIDENYL-
-0,657331407	METHOXAMINE HYDROCHLORIDE
-0,657868918	RILUZOLE
-0,65845466	CLOMIPRAMINE HYDROCHLORIDE
-0,658665155	CYPROHEPTADINE HYDROCHLORIDE

-0,65972211	CYCLOSPORINE
-0,660334997	BIFONAZOLE
-0,668497509	TRICLABENDAZOLE
-0,670580109	GABAPENTIN
-0,671535296	ACETYLTRYPTOPHAN
-0,675643697	NEOHESPERIDIN DIHYDROCHALCONE
-0,675658791	DIPLOSALSALATE
-0,678796667	AMSACRINE
-0,679231317	AGARIC ACID
-0,680377873	HAEMATOMMIC ACID, ETHYL ESTER
-0,68170306	COTARNINE CHLORIDE
-0,687214931	SALIDROSIDE
-0,687571658	STICTIC ACID
-0,68820809	SARAFLOXACIN HYDROCHLORIDE
-0,689170049	CILOSTAZOL
-0,691656799	CHOLESTANE
-0,695345271	VINCRISTINE SULFATE
-0,695945977	CHRYSIN
-0,696031115	BUSPIRONE HYDROCHLORIDE
-0,696318928	AMOXAPINE
-0,697350822	TRIFLURALIN
-0,699571658	PHTHALYLSULFACETAMIDE
-0,705758652	TRICHLORFON
-0,706731192	RISEDRONATE SODIUM HYDRATE
-0,70677101	ARTENIMOL
-0,707279323	BISOPROLOL FUMARATE
-0,70891157	CRYPTOTANSHINONE
-0,715019858	CHAULMOOGRIC ACID, ETHYL ESTER
-0,721741216	CANRENONE
-0,723845798	CHRYSOPHANOL
-0,724537183	PARAROSANILINE PAMOATE
-0,725817092	ANDROSTA-1,4-DIEN-3,17-DIONE
-0,730819203	QUEBRACHITOL
-0,732821789	ARIPIPRAZOLE
-0,735348351	ASPARTAME
-0,744183348	METHIMAZOLE
-0,744512951	CARBOFURAN
-0,748103594	FAMPROFAZONE
-0,758264724	TRICHLORMETHINE
-0,765729009	ASTEMIZOLE
-0,769470642	ARSENIC TRIOXIDE
-0,77178683	N-ACETYLPROLINE
-0,772139053	CHLOROGUANIDE HYDROCHLORIDE
-0,772487494	PREGABALIN
-0,774468272	ALRESTATIN
-0,775307259	DERRUSNIN
-0,785371483	TANSHINONE IIA
-0,787018662	PIPERIC ACID
-0,787833109	8-CYCLOPENTYLTHEOPHYLLINE
-0,789987742	LEUCOVORIN CALCIUM
-0,80099155	METHYL 7-DESHYDROXYPYROGALLIN-4-CARBOXYLATE
-0,806586274	ZILEUTON

-0,820760666	METHOXYAMINE HYDROCHLORIDE
-0,843399396	PIMETHIXENE MALEATE
-0,847902098	ISOBERGAPTENE
-0,852128108	CYTIDINE
-0,853509434	N-METHYLISOLEUCINE
-0,861470474	ACARBOSE
-0,865628996	OCTOPAMINE HYDROCHLORIDE
-0,867648927	NIALAMIDE
-0,868612763	ACECAINIDE HYDROCHLORIDE
-0,873810853	DIGITOXIN
-0,876416271	PROTRYPTYLINE HYDROCHLORIDE
-0,879162159	BENZETHONIUM CHLORIDE
-0,880344904	FURALTADONE
-0,887686269	TOREMIPHENE CITRATE
-0,9090411	DARIFENACIN HYDROBROMIDE
-0,925853539	DIMETHYL 4,4-o-PHENYLENE-BIS (3-THIOPHANATE)
-0,928432292	PROPARGITE
-0,933523242	CETRIMONIUM BROMIDE
-0,934704933	ISOPIMPINELLIN
-0,939224487	CAFESTOL ACETATE
-0,940546099	DIHYDROSTREPTOMYCIN SULFATE
-0,955711816	1-MONOPALMITIN
-0,960476082	ISORESERPINE
-0,96219849	ANTIMYCIN A (A1 shown)
-0,963442183	CORTISONE
-0,970980802	PHENOXYBENZAMINE HYDROCHLORIDE
-0,971564285	PRAVASTATIN SODIUM
-0,97301523	CLEMASTINE
-0,997479526	MIANSERIN HYDROCHLORIDE
-1,014108682	BROMPERIDOL
-1,01969221	ACETAMINOSALOL
-1,021689464	MECHLORETHAMINE
-1,028676231	LEVODOPA
-1,040040642	FUMARPROTOCETRARIC ACID
-1,041492535	3-AMINOPROPANESULPHONIC ACID
-1,055669546	DIHYDROJASMONIC ACID, METHYL ESTER
-1,06054934	EPIGALLOCATECHIN 3,5-DIGALLATE
-1,071544065	HOMIDIUM BROMIDE
-1,092699608	VESAMICOL HYDROCHLORIDE
-1,114103352	4-NONYLPHENOL
-1,121852305	DIHYDROROTENONE
-1,131167437	ISOROTENONE
-1,132070745	CHOLESTERYL ACETATE
-1,141189725	DIHYDROERGOTAMINE MESYLATE
-1,148269545	CEFAZOLIN SODIUM
-1,173372314	PERICIAZINE
-1,182558574	HEPTACHLOR
-1,199155502	METHYLBENZETHONIUM CHLORIDE
-1,20949101	GITOXIGENIN
-1,26797347	BAEOMYCESIC ACID
-1,268936133	PHENELZINE SULFATE
-1,285303482	HIERACIN

-1,293416593	DIETHYLCARBAMAZINE CITRATE
-1,302833316	MUNDULONE
-1,307838113	PARACHLOROPHENOL
-1,310031689	SODIUM THIOGLYCOLATE
-1,313973167	MECLIZINE HYDROCHLORIDE
-1,34447973	METHENAMINE
-1,350577508	NICOTINE DITARTRATE
-1,354632316	CLINDAMYCIN HYDROCHLORIDE
-1,359825488	METHICILLIN SODIUM
-1,360271467	NEOSTIGMINE BROMIDE
-1,37945454	DACTINOMYCIN
-1,388161963	DIGOXIN
-1,393460673	D-PHENYLALANINE
-1,445561865	PHENINDIONE
-1,514244396	GAMBOGIC ACID
-1,568112287	PENICILLIN G POTASSIUM
-1,577161779	BENZALKONIUM CHLORIDE
-1,596546472	PENICILLAMINE
-1,680021647	PENICILLIN V POTASSIUM
-1,846394091	GLYBURIDE
-1,864951683	PODOFILOX
-1,947457537	MECLOFENAMATE SODIUM
-2,091083381	PHENIRAMINE MALEATE
-2,214330239	PHENACEMIDE
-2,405699139	PARGYLINE HYDROCHLORIDE
-2,491230136	5alpha-CHOLESTAN-3beta-OL-6-ONE

Supplementary Table 2. List of first 18 hits confirmed by counterscreening and by qPCR on luciferase mRNA levels (Fig..xxxx)					
**Z score**	**MOLENAME**	**cas#**	**Formula**	**MolWt**	**Bioactivity**
11,55294	AUROTHIOGLUCOSE	12192-57-3	C6H11AuO5S	392,18257	antirheumatic
8,3054107	COLISTIMETHATE SODIUM	8068-28-8, 21362-08-3 [replaced]	C57H103N16Na5O28S5	1735,81586	antibacterial
9,0488367	DEXAMETHASONE	50-02-2	C22H29FO5	392,47183	glucocorticoid
9,5774494	COLCHICINE	64-86-8	C22H25NO6	399,44765	antimitotic, antigout agent
13,401401	CHLOROQUINE DIPHOSPHATE	54-05-7	C18H32ClN3O8P2	515,87164	antimalarial, antiamebic, antirheumatic, intercalating agent
7,6245709	CORTISONE ACETATE	50-04-4, 53-06-5 [cortisone]	C23H30O6	402,49195	glucocorticoid
9,4577015	FLUMETHAZONE PIVALATE	2002-29-1, 2135-17-3 [flumethasone]	C27H36F2O6	494,58117	glucocorticoid, antiinflammatory
9,5337825	ESTRADIOL VALERATE	979-32-8	C23H32O3	356,50969	estrogen
12,458741	ESTRIOL	50-27-1, 514-68-1 [as, succinate]	C18H24O3	288,39018	estrogen
7,7844529	ERGONOVINE MALEATE	129-51-1, 60-79-7 [ergonovine]	C23H27N3O6	441,48814	oxytocic, 5HT antagonist
8,9847892	NITROFURANTOIN	67-20-9, 54-87-5 [nitrofurantoin sodium], 17140-81-7 [monohydrate]	C8H6N4O5	238,16082	antibacterial
14,659134	MEGESTROL ACETATE	595-33-5, 3562-63-8 [megestrol]	C24H32O4	384,52024	progestogen, antineoplastic
7,1601434	MELPHALAN	148-82-3	C13H18Cl2N2O2	305,20661	antineoplastic, alkylating agent
7,0808647	METRONIDAZOLE	443-48-1, 69198-10-3 [metronidazole hydrochloride]	C6H9N3O3	171,15693	antiprotozoal
8,3633183	THIMEROSAL	54-64-8	C9H9HgNaO2S	404,81468	antiinfective, preservative
11,658503	DOXORUBICIN	23214-92-8	C27H29NO11	543,53228	antineoplastic
21,999416	DERRUBONE	22044-58-2	C21H18O6	366,37401	HSP90 inhibitor
14,847208	CYPROTERONE ACETATE	427-51-0	C24H29ClO4	416,94933	antiandrogen
